# A Sharp Quantitative Alexandrov Inequality and Applications to Volume Preserving Geometric Flows in 3D

**DOI:** 10.1007/s00205-025-02141-9

**Published:** 2025-11-21

**Authors:** Vesa Julin, Massimiliano Morini, Francesca Oronzio, Emanuele Spadaro

**Affiliations:** 1 Matematiikan ja Tilastotieteen Laitos, Jyväskylän Yliopisto, Jyväskylän, Finland; 2 Dipartimento di Scienze Matematiche Fisiche e Informatiche, Università di Parma, Parma, Italy; 3 Institutionen för Matematik, Kungliga Tekniska Högskolan, Stockholm, Sweden; 4 Dip. di Matematica, Univ. Roma-I “La Sapienza”, Roma, Italy

## Abstract

We study the asymptotic behavior of the volume preserving mean curvature and the Mullins–Sekerka flat flow in three dimensional space. Motivated by this, we establish a 3D sharp quantitative version of the Alexandrov inequality for $C^2$-regular sets with a perimeter bound.

## Introduction

In this paper we continue our study on the long-time behavior of globally well-defined weak solutions of two physically relevant volume preserving geometric flows in three dimensions: the volume preserving mean curvature and the Mullins–Sekerka flat flow. Our starting point is the work [25], which implies the qualitative convergence of the volume preserving mean curvature flat flow in $\mathbb {R}^3$ to a union of disjoint balls, up to translation of the components. In our previous work [24] on the two-dimensional case, we observed that the full quantitative convergence is related to a sharp quantitative version of the Alexandrov theorem, which is a purely geometric inequality. In [24] we proved a sharp quantitative Alexandrov theorem and show that it not only implies the full convergence of both the area preserving mean curvature and the Mullins–Sekerka flat flow, but also gives the exponential rate of convergence, in the planar case. Our main results in this paper are the three dimensional counterpart of both this inequality and its consequences for the asymptotics of the aforementioned geometric flat flows.

### Quantitative Alexandrov Inequality

We denote by $\textrm{H}_E$ the mean curvature of the hypersurface $\partial E$ (with the sign convention that $\textrm{H}_E\ge 0$ if *E* is convex, see the next section for details), *P*(*E*) for the perimeter and set that

$$ \bar{\mathrm {{H}}}_E := \frac{1}{P(E)}\int _{\partial E} \textrm{H}_E\, d\mathcal {H}^2. $$

Our first main results is as follow:

#### Theorem 1.1

For every $\delta _0>0$ there exists $C=C(\delta _0)>0$ such that, for every $C^2$-regular set $E\subset \mathbb {R}^3$ with

1.1 $$\begin{aligned} |E|=|B_1| \quad \text {and}\quad P(E) \le 4 \pi \, \root 3 \of {2} -\delta _0, \end{aligned}$$

it holds that

1.2 $$\begin{aligned} P(E)-P(B_1)\le C \, \big \Vert \textrm{H}_E-\bar{\mathrm {{H}}}_E\big \Vert _{L^2(\partial E)}^2. \end{aligned}$$

Moreover, there exists $\delta _1>0$ such that, if in addition to (1.1), $\partial E\in C^\infty$ and $\Vert \textrm{H}_E-\bar{\mathrm {{H}}}_E\Vert _{L^2(\partial E)}\le \delta _1$, then $\partial E$ is diffeomorphic to the standard sphere $\mathbb {S}^2$.

Let us make some comments on the result. The inequality (1.2) is a Łojasiewicz–Simon inequality for the perimeter functional with optimal exponent 2: indeed, (1.2) bounds the perimeter with the sharp power of the $L^2$-norm of its first variation, which is the mean curvature. We also call (1.2) a quantitative Alexandrov inequality because when the right hand side is zero, i.e., when $\partial E$ has constant mean curvature, then $P(E) \le P(B_1)$, which by the isoperimetric inequality means that *E* is the ball. This is the famous Alexandrov theorem and (1.2) is a sharp quantitative version of it, in the sense that the exponent two on the right hand side is optimal. Indeed, (1.2) is false for any exponent higher than two, see [24, Remark 2.2]. The second condition in the assumptions (1.1) is necessary, since the inequality (1.2) obviously fails for union of two balls with equal radius, and moreover, the constant *C* in (1.2) degenerates when $\delta _0 \rightarrow 0^+$. In order to see this, one may consider a union of two balls $E_\delta =B_{(1/2+\delta )^{1/3}}((-1,0,0))\cup B_{(1/2-\delta )^{1/3}}((1,0,0))$ and let $\delta \rightarrow 0$.

There has been an increasing interest on generalizations and quantifications of the Alexandrov theorem in recent years. We refer to [8] for an overview of this challenging problem, and mention the works [13–15] on the characterization of critical sets of the isoperimetric problem and [9, 10, 30] on quantification of the Alexandrov theorem. The main issue in the problem in Theorem 1.1 is that we do not have any a priori estimates, such as curvature bounds, for the set *E* other than the perimeter bound in (1.1), which prevents the bubbling phenomenon (concerning this point see [55]). Moreover, we measure the distance of the mean curvature to a constant with respect to $L^2$-norm, which in three dimension is not enough to apply Allard regularity theorem to deduce a priori regularity estimates for the set *E*. This makes the problem much more challenging than the two dimensional case. However, the inequality (1.2) is suitable for the study of the geometric equations, which was our main motivation to consider the problem in the first place.

We note that the right hand side (1.2) in $\mathbb {R}^3$ is special, since it is scaling invariant. The novelty of the paper is that we prove the inequality (1.2) by means of results from conformal geometry, by transforming the problem into a minimization problem involving Canham–Helfrich energy functional. Indeed, using the estimates from [25] we are able to restate the problem using parametizations by weak immersions from $\mathbb {S}^2$ to $\mathbb {R}^3$, which allows us to use the results [46, 47, 56] to obtain the existence and regularity of the minimizer of an auxiliary problem (see Proposition 4.1). We then derive uniform regularity estimates which enable us to prove (1.2).

The closest result to Theorem 1.1 in the literature is the quantitative Willmore inequality due to Röger and Schätzle [58], which roughly states that, for *E* as in Theorem 1.1 and $\partial E$ diffeomorphic to the sphere, it holds that

1.3 $$\begin{aligned} P(E)-P(B_1)\le C( \Vert \textrm{H}_E \Vert _{L^2(\partial E)}^2 - 16 \pi ). \end{aligned}$$

However, the inequality (1.2) is stronger than (1.3) (in the sense that (1.2) implies (1.3)) and (1.3) is not enough for the applications to geometric flows. Moreover, the proof of (1.3) is completely different to (1.2), as it relies on the quantitative result on nearly umbilical sets due to De Lellis and Müller [11].

### Application I: Volume Preserving MCF

As we already mentioned, our main motivation to prove Theorem 1.1 is to study the asymptotic behavior of volume preserving geometric flows. We begin with the *volume preserving mean curvature flow* and recall that a smooth flow of sets $(E_t)_{t\in [0,T)}\subset \mathbb {R}^3$, for some $T>0$, is a solution to the volume preserving mean curvature flow if it satisfies that

1.4 $$\begin{aligned} V_t = -\textrm{H}_{E_t} + \bar{\mathrm {{H}}}_{E_t} \quad \text {on }\partial E_t\subset \mathbb {R}^3, \end{aligned}$$

where $V_t$ denotes the outer normal velocity. Such a geometric flow has been proposed in the physical literature as a model for coarsening phenomena. We refer to [7, 49, 64, 65] for an introduction to the physical background. For us the most important feature is that (1.4) can be seen as a gradient flow of the perimeter with respect to a suitable (formal) $L^2$-type Riemannian structure and it preserves the volume.

In general smooth solutions of (1.4) may develop singularities in finite time and therefore we need a suitable notion of weak solution which is defined for all times. The main difference between (1.4) and the mean curvature flows is that (1.4) is non-local and does not satisfy the comparison principle, and therefore it is not clear how to define a level set solution to it. A well established choice for a weak solution of (1.4) is the minimizing movement approach proposed for the mean curvature flow independently by Almgren, Taylor and Wang [3] and by Luckhaus and Stürzenhecker [40], and adapted to the volume constrained case in [50]. Here we use the definition from [23] as it simplifies the analysis. We recall that the minimizing movement method is based on the gradient flow structure of the flow. To be more precise, one uses implicit time-discretization and recursive minimization of a suitable incremental problem to construct a discrete-in-time approximation of a solution to the equation (1.4) and defines any of its cluster points, as the time step converges to zero, as *a flat flow* solution of (1.4). For the precise definition see Section 5. By the results in [3, 40, 50] the flat flow, starting from any bounded set of finite perimeter, always exists and is Hölder continuous in the $L^1$-topology. Moreover by the result in [26] it coincides with the unique smooth solution as long as the latter exists. For other possible notions of weak solutions we refer to [27] for a viscosity solution for star-shaped sets, and the gradient flow calibration in [35]. We also mention the recent works on the phase-field approximation to (1.4) via the Allen-Cahn equation, which converges to a Brakke-type solution of (1.4) [62, 63], to a distributional solution if the convergence is assumed to be strong in BV by [36], and the consistency is proven in [29].

Since the flat flow is defined globally in time we may study its asymptotics. Apart from our previous work [24] that we already mentioned, the long-time convergence results in the literature are confined to the case when the classical solution is defined for all times, e.g., when the initial set is convex [19], or when the initial set is close to the ball [16] or to a general local minimum in the case of a flat torus [12, 51]. The starting point of our analysis is the result by the first author and Niinikoski [25] on the qualitative asymptotic convergence of (1.4), which is stated as follows: given any volume preserving flat flow $\{E(t)\}_{t\ge 0}$ in $\mathbb {R}^3$ starting from a bounded set of finite perimeter $E(0)\subset \mathbb {R}^3$ with volume $|E(0)| = v$, there exist $N \in \mathbb {N}$ and time-dependent points

1.5 $$\begin{aligned} x_1(t), \dots , x_N(t) \in \mathbb {R}^3, \quad |x_i(t) -x_j(t)| \ge 2r\quad \forall \;i\ne j \end{aligned}$$

such, that setting $r = \left( \frac{3 v}{4 \pi N}\right) ^{1/3}$ and $F(t) = \bigcup _{i =1}^N B_r(x_i(t)),$ we have that

1.6 $$\begin{aligned} \sup _{x\in E(t) \Delta F(t)} dist (x, \partial F(t)) \rightarrow 0 \quad \text {as } \, t \rightarrow \infty . \end{aligned}$$

We remark that it is possible that the flow converges to a union of tangent balls [17], but it is expected that this happens only in special cases, and that generically the limiting set is a unique ball. Based on this, we show that if in the above theorem, the set *F*(*t*) is just one ball, or if the balls have positive distance to each other, then we may upgrade the above convergence result to a full quantitative exponential convergence to a union of (independent of time) balls. In both cases, the crucial estimate is given by Theorem 1.1. We prove the following result:

#### Theorem 1.2

Let $\{E(t)\}_{t\ge 0}$, $N \in \mathbb {N}$ and the points $x_1(t), \dots , x_N(t)$ in $\mathbb {R}^3$ be as (1.5) above and assume in addition that there exists $\delta _1>0$ and $T_0$ such that

1.7 $$\begin{aligned} |x_i(t) -x_j(t)| \ge 2r + \delta _1 \quad \text { for all } \, t \ge T_0 \end{aligned}$$

for $i \ne j$. Then the sets *E*(*t*) converge exponentially fast to $F = \bigcup _{i =1}^N B_r(x_i)$, for some $x_1,\dots , x_N\in \mathbb {R}^3$ with $|x_i -x_j| >2r$, $i \ne j$. To be more precise, we have

$$ \sup _{x\in E(t) \Delta F} dist (x, \partial F) + |P(E(t)) - N 4 \pi r^2 | \le C e^{-\frac{t}{C}} $$

for a constant $$C>1$$.

In particular, if the set *F*(*t*) in (1.6) is just one ball, then by Theorem 1.2 we have the full convergence of the flow. For example, if the initial set satisfies the condition (1.1), i.e., $|E(0)| = |B_1|$ and $P(E(0))\le 4 \pi \, \root 3 \of {2} -\delta _0$, or if there is $T>0$ such that *E*(*t*) satisfies the condition (1.1) for all $t \ge T$, then the flow converges exponentially fast to a ball. We recall that we do not need any assumptions on the regularity, other than *E*(0) being set of finite perimeter, or topological assumptions on the initial set.

### Application II: Mullins–Sekerka Flow

The second geometric evolution equation that we consider, is the two-phase Mullins–Sekerka flow. We will define the flow in the three dimensional flat torus of side length *R*, and assume *R* large, instead of considering a bounded domain to avoid boundary effects. We choose the side length to be large with respect to the volume of the set, to rule out other possible limit sets than union of balls. We note that the isoperimetric problem for a general volume in the flat torus is a well-known open problem. The Mullins–Sekerka flow is then given by the dynamics

1.8 $$\begin{aligned} {\left\{ \begin{array}{ll} V_t = [\partial _\nu u_{E(t)}] \quad \text {on } \, \partial E(t)\\ \Delta u_{E(t)} = 0 \quad \text {in } \, \mathbb {T}_R^3 \setminus \partial E(t)\\ u_{E(t)} = H_{E(t)} \quad \text {on } \, \partial E(t), \end{array}\right. } \end{aligned}$$

where $[\partial _\nu u_{E(t)}]$ denotes the jump of the normal.

Similar to (1.4), also the Mullins–Sekerka flow is a non-local, volume preserving mean curvature flow. It can also be seen as a gradient flow of the surface area but this time with respect to a suitable $H^{-\frac{1}{2}}$-Riemannian structure. It can be seen as a quasistatic variant of the Stefan problem [39] and as a singular limit of the Cahn-Hilliard equation, see [2, 53]. For a more comprehensive introduction we refer to [24] and the reference therein.

There is no result similar to (1.6) for the Mullins–Sekerka flow in the literature. Therefore we need first to prove similar qualitative convergence for (1.8), and after that we may prove the exponential convergence similar to Theorem 1.2. Below is our result for the asymptotical behavior of (1.8).

#### Theorem 1.3

Fix $v, M>0$. Then there exists $R_0 \ge 1$, depending on *v* and *M*, with the following property: Let $\{E(t)\}_{t\ge 0}$ be a flat flow for (1.8) in $\mathbb {T}_R^3$, $R \ge R_0$, starting from a set of finite perimeter $E(0)\subset \mathbb {T}_R^3$ with volume $|E(0)| = v$ and $P(E(0)) \le M$. There exist $N \in \mathbb {N}$ and time-dependent points $x_1(t), \dots , x_N(t)$ in $\mathbb {T}_R^3$ such that setting $r = \left( \frac{3 v}{4 \pi N}\right) ^{1/3}$ and $F(t) = \bigcup _{i =1}^N B_r(x_i(t))$ it holds $|x_i(t) -x_j(t)| \ge 2r$, $i \ne j$,

$$ | E(t) \Delta F(t)| \rightarrow 0 \quad \text {as } \, t \rightarrow \infty . $$

Assume, in addition, that there exists $\delta _1>0$ and $T_0$ such that

$$ |x_i(t) -x_j(t)| \ge 2r + \delta _1 \quad \text { for all } \, t \ge T_0 $$

for $i \ne j$. Then, the sets *E*(*t*) converge exponentially fast to a (independent of time) union of balls

$$ F = \bigcup _{i =1}^N B_r(x_i),\quad \text { where }\;|x_i -x_j| >2r\quad i \ne j. $$

More precisely, there exists a constant $C>1$ such that

$$ |E(t) \Delta F| + |P(E(t)) - N 4 \pi r^2 | \le C e^{-\frac{t}{C}}. $$

As in the two-dimensional case, in addition to Theorem 1.1 we need also the density estimates due to Schätzle [60] in order to prove a quantitative Alexandrov theorem in terms of the potential $u_{E(t)}$, see Proposition 6.3. We remark that we expect the convergence in Theorem 1.3 to hold also with respect to the Hausdorff distance, as is the case in Theorem 1.2. Finally we remark that the convergence of so called distributional solution to the half-space in $\mathbb {R}^n$ is considered in [52]; it is interesting that in that case the convergence is not exponential.

## Notation and Preliminaries

We begin with the notation related to the Euclidian space. We denote the open ball with radius *r* centered at *x* by $B_r(x)$ and by $B_r$ if it is centered at the origin. For $X \in C^1(U;\mathbb {R}^n)$, $U \subset \mathbb {R}^n$ open, we denote its differential by *DX* and the divergence by $\textrm{div}X = \text {Tr}(DX)$. Similarly for a real valued function $u \in C^2(U)$ we denote the gradient by *Du*, the Hessian by $D^2 u$ and the Laplacian by $\Delta u$. We point out that we will use the symbol $\nabla$ for the covariant derivative on a manifold, which we will introduce shortly. However, with a slight abuse of notation we will still use the symbols $\Delta$ and $''\textrm{div}''$ for the Laplace-Beltrami operator and the divergence on a manifold respectively. The use will be clear from the context.

Given a set $E \subset \mathbb {R}^n$, we define the distance function $dist (\cdot , E):\mathbb {R}^n\rightarrow [0,+\infty )$, as usual $dist (x, E)= \inf _{y\in E}|x-y|$, and the signed distance function $d_E:\mathbb {R}^n\rightarrow \mathbb {R}$ as

2.1 $$\begin{aligned} d_E(x) = dist (x, E) - dist (x, \mathbb {R}^n \setminus E)\,. \end{aligned}$$

Then clearly it holds that $dist (\cdot , \partial E) =|d_E|$. For any Lebesgue measurable set $E \subset \mathbb {R}^n$ we denote by |*E*| its Lebesgue measure and define the perimeter of *E* in an open set $U\subset \mathbb {R}^n$ as

2.2 $$\begin{aligned} P(E,U)=\sup \Big \{\int _{E}\textrm{div}X \,dx\,:\, X\in C_0^1(U,\mathbb {R}^n)\,\,\,\text {with}\,\,\,\Vert X\Vert _{L^{\infty }}\le 1\Big \}\,. \end{aligned}$$

We write that $P(E)= P(E,\mathbb {R}^n),$ and if $P(E)<+\infty$, we say that *E* is a set of finite perimeter. In this case, the reduced boundary of *E* is denoted by $\partial ^*E$, and the unit outer normal to *E* by $\nu _E$. Then, $P(E, U) = \mathcal {H}^{n-1}(\partial ^* E\cap U)$ for any open set *U*. For a given vector field $X \in C^1(\mathbb {R}^n;\mathbb {R}^n)$ we define its tangential differential on $\partial E$ by $\nabla _{T} X = D X - (D X \nu _E) \otimes \nu _E$ and tangential divergence as $\textrm{div}^T X = \text {Tr}(\nabla _{T} X )$. If *E* is $C^2$-regular, i.e., $\partial E$ is $C^2$-hypersurface, we define the mean curvature $\textrm{H}_E$ as the sum of the principal curvatures and denote the second fundamental form by $\textrm{A}_E$. We use the orientation such that $\textrm{H}_E$ is nonnegative for convex sets. The divergence theorem extends to vector fields $X\in C^1(\mathbb {R}^n,\mathbb {R}^n)$ as

$$ \int _{\partial E} \textrm{div}^T X\,d\mathcal {H}^{n-1}= \int _{\partial E} \textrm{H}_E (X\cdot \nu _E)\,d\mathcal {H}^{n-1}, $$

where $\cdot$ is the inner product in $\mathbb {R}^n$. Recall that $\bar{\mathrm {{H}}}_E$ denotes the integral average of the mean curvature, i.e. $(\int _{\partial E}\textrm{H}_E\,d\mathcal {H}^{n-1})/ P(E).$ This notation is related to sets in $\mathbb {R}^n$. We will also need notation related to more general Riemann manifolds and introduce the notation related to this.

### Smooth Immersions

Let $\Sigma$ be a 2-dimensional, connected (unless otherwise specified), smooth manifold and consider a smooth immersion $\vec {f}: \Sigma \rightarrow \mathbb {R}^3$. In this work, we will call $\vec {f}(\Sigma )$ immersed surface and “smooth” always means of class $C^\infty$. In such a setting, $\Sigma$ is naturally endowed with a Riemannian metric, which is $g=\vec {f}^*g_{\mathbb {R}^3}$, given in local coordinates as $g_{ij}=\partial _{x^{i}}\vec {f}\cdot \partial _{x^{j}} \vec {f}$. The metric *g* can then be extended to tensors via the formula

$$\begin{aligned} g(T,S)=g_{i_{1}s_{1}} \dots g_{i_{k}s_{k}} g^{j_{1}z_{1}}\dots g^{j_{l}z_{l}} T^{ i_1...i_k}_{j_1...j_l} S^{s_1...s_k}_{z_1...z_l }, \end{aligned}$$

where $(g^{ij})$ is the inverse matrix of $(g_{ij})$. We note that in the above and throughout the paper we adopt the Einstein summation convention. The norm of a tensor *T* is then defined as $|T|=\sqrt{g(T,T)}$ and it satisfies the following useful properties:

2.3 $$\begin{aligned} |g(S,T)|\le |S|\,|T|\quad \quad \quad |S+T|\le |S|+|T|. \end{aligned}$$

One may define uniquely the area (or canonical) measure $\sigma$ on $\Sigma$ by imposing in any chart $(U,\varphi )$ that $\sigma (B)= \int _{\varphi (B)}\sqrt{\det g_{ij}}\circ \varphi ^{-1}\,dx$. The measure $\sigma$ is then a complete, regular Radon measure.

We denote by $\nabla$ the Levi-Civita connection of $(\Sigma ,g)$. We may extend $\nabla$ uniquely to every bundle of tensors (by defining it in a natural way on $C^{\infty }(\Sigma )$ and by imposing the Leibniz rule and the commutativity with any contraction). In local coordinates it is given by

$$\begin{aligned} \bigl (\nabla _{X}T\bigr )^{i_1\dots i_r}_{j_1\dots j_s}=X^{k}\biggl [\partial _{x^k}T^{i_1\dots i_r}_{j_1\dots j_s}-\sum _{p=1}^{s}\Gamma ^{l}_{kj_{p}}T^{i_1\dots i_r}_{j_1\dots j_{p-1} l\, j_{p+1}\dots j_s }+\sum _{q=1}^{r}\Gamma ^{i_{q}}_{kl}T^{i_1\dots i_{q-1}l\, i_{q+1}\dots i_r}_{j_1\dots j_s}\biggr ], \end{aligned}$$

where Christoffel symbols $\Gamma _{ij}^k$ are expressed in terms of the coefficients of the metric *g* as

$$\begin{aligned} \Gamma _{ij}^k=\frac{g^{kl}}{2}\left( \partial _{x^i} g_{lj} + \partial _{x^j}g_{il}- \partial _{x^l}g_{ij}\right) . \end{aligned}$$

We will write $\nabla ^mT$ for the *m*-th iterated covariant derivative of *T* and the formula for the interchange of covariant derivatives, which involves the Riemann tensor, is

2.4 $$\begin{aligned} \nabla ^{2}T_{abj_1\dots j_s}^{i_1\dots i_r}-\nabla ^{2}T_{baj_1\dots j_s}^{i_1\dots i_r}&=\sum _{p=1}^{s}R_{abj_pm}g^{ml}T^{i_1\dots i_r}_{j_1\dots j_{p-1}l\, j_{p+1}\dots j_s }\nonumber \\&\quad +\sum _{q=1}^{r}R_{abml}g^{mi_q}T^{i_1\dots i_{q-1}l\, i_{q+1}\dots i_r}_{j_1\dots j_s}, \end{aligned}$$

where we recall that

$$\begin{aligned} R_{ijkl}=\Bigl (\partial _{x^j}\Gamma _{ik}^m-\partial _{x^i}\Gamma _{jk}^m+\Gamma _{ik}^s\Gamma _{js}^m-\Gamma _{jk}^s\Gamma _{is}^m\Bigr )g_{ml}. \end{aligned}$$

Finally, the (rough) Laplacian $\Delta T$ of a tensor *T* is defined as $g^{ij}\nabla ^2T_{ij}$.

Let $\vec {f}: \Sigma \rightarrow \mathbb {R}^3$ be a smooth immersion of a generic connected smooth 2-manifold $\Sigma$. Since $d\vec {f}_p:T_p\Sigma \rightarrow \mathbb {R}^3$ is an injective linear map, we identify $T_p\Sigma$ with the linear subspace $d\vec {f}_p(T_p\Sigma )$ of $\mathbb {R}^3$, for all $p\in \Sigma$, and by virtue of this identification, at every point $p \in \Sigma$ we can define up to a sign a unit normal vector $\nu (p)$. Let us observe that if $\Sigma$ is closed and $\vec {f}$ is also injective, then the unit normal vector $\nu (p)$ can be chosen so that it depends globally in a $C^\infty$-way on the point *p*. Indeed, if $\Sigma$ is closed and $\vec {f}$ is also injective, then $\vec {f}$ is an embedding and $\vec {f}(\Sigma )$ admits a unique smooth structure making it into an embedded surface of $\mathbb {R}^3$ with the property that $\vec {f}$ is a diffeomorphism onto its image. Hence, $\vec {\iota }:\vec {f}(\Sigma )\rightarrow \mathbb {R}^3$ is a smooth embedding, the cited smooth structure on $\vec {f}(\Sigma )$ is determined by the smooth atlas formed by the charts $( \vec {f}(U), \varphi \circ {\vec {f}}^{\,\,-1})$, where $(U,\varphi )$ is any chart of $\Sigma$, and in this case, the tangent spaces $T_{\vec {f}(p)} \vec {f}(\Sigma )$ and $T_p\Sigma$ are identified with the same linear subspace of $\mathbb {R}^3$. Then, by the theorem of Jordan-Brouwer [5, Proposition 12.2], $\mathbb {R}^3\setminus \vec {f}(\Sigma )$ has two connected components and one of them is a compact smooth 3-dimensional submanifold of $\mathbb {R}^3$ with boundary $\vec {f}(\Sigma )$. Accordingly, there exists ${\bar{\nu }}:\vec {f}(\Sigma )\rightarrow \mathbb {R}^3$ global unit normal smooth vector field *pointing outward*, as well as ${\bar{N}}:\vec {f}(\Sigma )\rightarrow \mathbb {R}^3$ global unit normal smooth vector field *pointing inward*, ${\bar{N}}=-{\bar{\nu }}$. Thus, $\nu ={\bar{\nu }} \circ \vec {f}$ and $N={\bar{N}}\circ \vec {f}$ will be the outward-pointing and inward-pointing global unit normal smooth vector field of $\Sigma$, respectively. Therefore, $\Sigma$ is orientable. In fact, the theorem of Jordan-Brouwer and relative consequences are in general true for every closed, connected, embedded smooth surface ${\bar{\Sigma }}$ of $\mathbb {R}^3$. The surface ${\bar{\Sigma }}$ is orientable, since an orientation of ${\bar{\Sigma }}$, which will be considered from now on, is determined by the charts $({\bar{U}},{\bar{\varphi }}=(x^1,x^2))$ of ${\bar{\Sigma }}$ for which

2.5 $$\begin{aligned} {\bar{N}}_p\,=\,\frac{\partial _{x^{1}}\times \partial _{x^{2}}}{|\partial _{x^{1}}\times \partial _{x^{2}}|}(p) \,=\frac{\partial _{x^{1}}{\bar{\psi }}\times \partial _{x^{2}}{\bar{\psi }}}{\big [\,|\partial _{x^{1}}{\bar{\psi }}|^2\,|\partial _{x^{2}}{\bar{\psi }}|^2 \,-\,(\partial _{x^{1}}\bar{\psi }\cdot \partial _{x^{2}}{\bar{\psi }})^2\,\big ]^{1/2}}\,({\bar{\varphi }}(p)) \end{aligned}$$

for every $p\in {\bar{U}}$, where ${\bar{\psi }}={\bar{\varphi }}^{-1}$ and ${\bar{N}}:{\bar{\Sigma }}\rightarrow \mathbb {R}^3$ is global unit normal smooth vector field pointing inward, by noting that the Jacobian determinant of the transition maps between of two charts of ${\bar{\Sigma }}$, which satisfy the identity (2.5), is positive. In the case ${\bar{\Sigma }}=\vec {f}(\Sigma )$, then we obtain the existence of an atlas $\mathcal {A}=\{\big (U_i,\varphi _i=(x^1,x^2)\big )\}_{i\in I}$ of $\Sigma$ such that both

2.6 $$\begin{aligned} N_p={\bar{N}}_{\vec {f}(p)}\,=\,\frac{\partial _{x^{1}} ( \vec {f}\circ \varphi _i^{-1}) \times \partial _{x^{2}} ( \vec {f}\circ \varphi _i^{-1})}{|\partial _{x^{1}}( \vec {f}\circ \varphi _i^{-1})\times \partial _{x^{2}} ( \vec {f}\circ \varphi _i^{-1})|}\,(\varphi _i(p))\,=\,\frac{\partial _{x^{1}} \vec {f} \times \partial _{x^{2}}\vec {f}}{|\partial _{x^{1}} \vec {f} \times \partial _{x^{2}} \vec {f}|}\,(p) \end{aligned}$$

for all $p\in U_i$ and every $i\in I$, and the Jacobian determinants of all its transition maps are positive, hence $\Sigma$ is orientable and its orientation is that one given by the atlas $\mathcal {A}$.

Let $\vec {f}: \Sigma \rightarrow \mathbb {R}^3$ be a smooth immersion of a connected, 2-dimensional, smooth manifold $\Sigma$. As in the case of sets, we may define the second fundamental form and the mean curvature also in this case. However, we prefer to keep the notation from differential geometry as it is standard in this context. For a choice of the smooth unit normal vector field $\nu$ around a point $p\in \Sigma$, the (scalar) second fundamental form at *p* is the symmetric bilinear form on $T_p\Sigma$ defined as

2.7 $$\begin{aligned} \textrm{A}_p(v,w)= \overline{\nabla }_v \nu \cdot w= N\cdot \overline{\nabla }_v w \end{aligned}$$

for all $v, w \in T_p\Sigma$, where $N=-\nu$ and $\overline{\nabla }$ is the Levi-Civita connection of $(\mathbb {R}^3, g_{\mathbb {R}^3})$. Then we have the (scalar) mean curvature at *p*, $\textrm{H}_p$, which is the trace of $\textrm{A}_p$ with respect $g_p$ (that is $\textrm{H}_p=\textrm{A}_p(e_1,e_1)+\textrm{A}_p(e_2,e_2)$, for any orthonormal basis $\{e_1,e_2\}$ of $T_p\Sigma$), the vector mean curvature at *p*, $\vec {\textrm{H}}_p=\textrm{H}_pN=-\textrm{H}_p\nu$, the traceless part $\mathrm {\text{\AA }}_p$ of $\textrm{A}_p$, given by $\mathrm {\text{\AA }}_p= \textrm{A}_p - (\textrm{H}_p/2)g_p$, and the Weingarten operator at *p*, which is the linear map $\textrm{W}_p:T_p\Sigma \rightarrow T_p\Sigma$ defined through $g_p(\textrm{W}_p (v), w)=\textrm{A}_p (v,w)$ for all $v,w\in T_p\Sigma$. The eigenvalues $k_1\le k_2$ of $\textrm{W}_p$ are the principal curvatures at *p*, and we have the following identities: $|\textrm{A}|^2=k_1^2+k_2^2$, $|\mathrm {\text{\AA }}|^2=(k_2-k_1)^2/2$; $\textrm{H}=k_1+k_2$; $\textrm{K}^{G}=k_1k_2$. Here, $\textrm{K}^{G}$ is the Gaussian curvature, which is equal to the half of the scalar curvature of $(\Sigma ,g)$. Notice that locally, we may always choose $\nu$ to be smooth.

The Riemann tensor, the Ricci tensor and the scalar curvature can be expressed via the second fundamental form as

$$\begin{aligned} R_{ijkl}&=\textrm{A}_{ik}\textrm{A}_{jl}-\textrm{A}_{il}\textrm{A}_{jk}\\ \textrm{Ric}_{ij}&=g^{kl}R_{ikjl}=\textrm{HA}_{ij}-\textrm{A}_{il}g^{lk}\textrm{A}_{kj}\\ \textrm{Sc}&=g^{ij}\textrm{Ric}_{ij}=\textrm{H}^2-|\textrm{A}|^2 \end{aligned}$$

A consequence of this link between the Riemann tensor and the second fundamental form and of the formula for the interchange of the covariant derivatives (2.4) is the useful equality

2.8 $$\begin{aligned} \big (\Delta \nabla \textrm{H}-\nabla \Delta \textrm{H}\big )_i=\textrm{HA}_{ij}g^{jk}\nabla \textrm{H}_k-\textrm{A}_{ij}g^{jk}\textrm{A}_{kl}g^{lm}\nabla \textrm{H}_m. \end{aligned}$$

The symmetry properties of the covariant derivative of $\textrm{A}$, given by the Codazzi equations $\nabla \textrm{A}_{ijk}=\nabla \textrm{A}_{jik}=\nabla \textrm{A}_{kij}$, imply $\nabla \textrm{H}=\textrm{div A}=2\,\mathrm {div \text{\AA }}$, which in turn yield the inequalities

2.9 $$\begin{aligned} |\textrm{H}|^2&\le 2\,|\textrm{A}|^2,\quad \quad \quad |\nabla \textrm{H}|^2\le 2\,|\nabla \textrm{A}|^2\nonumber \\&\le 12\,|\nabla \mathrm {\text{\AA }}|^2, \quad \quad \quad |\nabla ^2\textrm{H}|^2\le 2\,|\nabla ^{2}\textrm{A}|^2\le 12\,|\nabla ^2\mathrm {\text{\AA }}|^2 \end{aligned}$$

and the well-known Simons’ identity

2.10 $$\begin{aligned} \Delta \textrm{A}_{ij} = \nabla ^2\textrm{H}_{ij} + \textrm{HA}_{il} g^{ls} \textrm{A}_{sj} - |\textrm{A}|^2 \textrm{A}_{ij}. \end{aligned}$$

The *Willmore energy* of a smooth immersion $\vec {f}: \Sigma \rightarrow \mathbb {R}^3$, for a generic connected, closed, smooth 2-manifold $\Sigma$, is given by

2.11 $$\begin{aligned} \mathcal {W}(\vec {f})=\frac{1}{4}\int _{\Sigma } \textrm{H}^2\,d\sigma \,. \end{aligned}$$

The most important property of this functional is its invariance under conformal diffeomorphisms of $\mathbb {R}^3$, recalling that a smooth immersion $f: (M, g)\rightarrow (N, h)$ between two Riemannian manifolds (*M*, *g*) and (*N*, *h*) is said to be conformal if $f^* h=e^{2\lambda }g$ for some $\lambda \in C^{\infty }(M)$. More precisely, $\mathcal {W}(\Phi \circ \vec {f})=\mathcal {W}(\vec {f})$, where $\Phi$ is any combination of a translation of $\mathbb {R}^3$ given by $x \mapsto x+x_0$ for a fixed $x_0\in \mathbb {R}^3$, a dilation of $\mathbb {R}^3$ defined as $x \mapsto \alpha x$ for some $\alpha >0$, and a spherical inversion $\mathbb {R}^3$ with center $p \notin \vec {f}(\Sigma )$ and radius $r>0$ described as $x \mapsto r^2 (x-p)/|x-p|^2$. In the case that $\Phi$ is a spherical inversion with center $p\in \vec {f}(\Sigma )$, we have $\mathcal {W}(\Phi \circ \vec {f})=\mathcal {W}(\vec {f})-4\pi \,\sharp \vec {f}^{-1}(p)$, where $\sharp (\cdot )$ is the cardinality of $(\cdot )$, [4]. As a consequence, we get the Li–Yau inequality [38], that is $\mathcal {W}(\vec {f}) \ge 4\pi \, \sharp \vec {f}^{-1}(p)$ for all $p\in \vec {f}(\Sigma )$. In particular if $\vec {f}$ is not an embedding, then $\mathcal {W}(\vec {f})\ge 8\pi$.

The infimum of $\mathcal {W}(\vec {f})$ among all smooth immersions $\vec {f}: \Sigma \rightarrow \mathbb {R}^3$, where $\Sigma$ is a connected, closed, smooth 2-manifold, belongs to $[4\pi ,8 \pi )$ if all 2-manifolds $\Sigma$ are orientable. More precisely, for each nonnegative integer $\mathfrak {g}$, setting that

$$\begin{aligned} \beta _\mathfrak {g}:=\inf \Big \{ \mathcal {W}(\vec {f})\,\,;\,&\, \vec {f}:\Sigma \rightarrow \mathbb {R}^3 \text{ is } \text{ a } \text{ smooth } \text{ immersion } \text{ with } \Sigma \text{ a } \text{2-dimensional, } \\&\,\text{ connected, } \text{ closed, } \text{ orientable, } \text{ smooth } \text{ manifold } \text{ of } \text{ genus } \mathfrak {g}\Big \}, \end{aligned}$$

it turns out $4\pi \le \beta _\mathfrak {g}<8\pi$ and each infimum $\beta _\mathfrak {g}$ is attained by a smooth embedding $\vec {F}:\Sigma \rightarrow \mathbb {R}^3$, [4, 37, 61, 66]. The equality $\beta _\mathfrak {g}=4\pi$ holds if and only if $\mathfrak {g}=0$ and $\vec {F}$ is a round sphere, that is, $\Sigma$ is topologically a sphere and $\vec {F}$ embeds $\Sigma$ as a round 2-sphere of $\mathbb {R}^3$. Similarly, $\beta _\mathfrak {g}\ge 2\pi ^2$ for any $\mathfrak {g}\ge 1$ and the equality holds if and only if $\mathfrak {g}=1$ and $(\pi ^{-1}\circ \vec {F})(\Sigma )$ is the Clifford torus $\mathbb {S}^1_{\frac{1}{\sqrt{2}}}\times \mathbb {S}^1_{\frac{1}{\sqrt{2}}}$, up to conformal diffeomorphisms of $\mathbb {S}^3$, [42], where $\mathbb {S}^n_r$ denotes the sphere of radius *r* and center the origin of $\mathbb {R}^{n+1}$ and $\pi$ is a generic stereographic projection of $\mathbb {S}^3$. For completeness, we mention that there exists the limit of the $\beta _\mathfrak {g}$’s for $\mathfrak {g}\rightarrow +\infty$ and it holds that $\lim _{\mathfrak {g}\rightarrow +\infty }\beta _\mathfrak {g}=8\pi$, [32].

### Embedded Surfaces and Riemann Surfaces

Given a closed connected embedded smooth surface $\Sigma \subset \mathbb {R}^{3}$, the inclusion $\vec {\iota }:\Sigma \rightarrow \mathbb {R}^3$ is a smooth immersion. Thus, the above is applicable, recovering in this way the classic theory of the embedded surfaces of $\mathbb {R}^3$. In particular, $\Sigma$ is a Riemannian surface where the metric *g* is the (natural) one induced by the Euclidian metric, it is orientable with orientation fixed through (2.5), and the second fundamental form $\textrm{A}$, the mean curvature $\textrm{H}$, the traceless part $\mathrm {\text{\AA }}$ of $\textrm{A}$ can be globally defined in a smooth way on it. In the case that the surface is the boundary of a set *E*, i.e. $\Sigma = \partial E$, we use the notation $\textrm{H}_E$, $\textrm{A}_E$, etc., while if we are interested only on the surface, we write $\textrm{H}_\Sigma , \textrm{A}_\Sigma$ etc..

Since $\Sigma$ is a closed connected orientable smooth 2-manifold, it is diffeomorphic either to a sphere or to a connected sum of tori, [6, 43]. If it is diffeomorphic to a sphere, we say that it has genus 0, while if it is diffeomorphic to the connected sum of *n* tori, we say that it has genus *n*. It follows then from Gauss-Bonnet theorem (see for instance [54]) that

2.12 $$\begin{aligned} \int _{\Sigma } \textrm{K}^G_{\Sigma }\,d\mathcal {H}^2=2\pi \chi (\Sigma )=4\pi \big (1-\text {genus}(\Sigma )\big ). \end{aligned}$$

Another important property for the surface $\Sigma$ is the existence of an atlas $\{\big (U_j, (u_j,v_j)\big )\}_{j\in J}$, compatible with the fixed orientation, such that all local coordinates $(u_j,v_j)$ are isothermal for the Riemannian metric *g*, i.e.,

$$\begin{aligned} g=e^{2\lambda _j}(du_j\otimes du_j+dv_j\otimes dv_j), \end{aligned}$$

with $\lambda _j\in C^{\infty }(U_j)$. Moreover, the family $\{(U_j, u_j+i v_j)\}_{j\in J}$ defines a one-dimensional complex structure on $\Sigma$, since all its transition maps and their inverses are holomorphic, and the Riemann surface *S* so obtained, that is the one-dimensional complex manifold determined by this complex structure, is said to be *induced by the Riemmanian oriented surface* $(\Sigma , g)$. If *S* and $S'$ are Riemann surfaces induced by the Riemmanian oriented surfaces $(\Sigma , g)$ and $(\Sigma ', g')$ respectively, then $f:(\Sigma , g)\rightarrow (\Sigma ', g')$ is a smooth orientation-preserving conformal diffeomorphism if and only if $f:S\rightarrow S'$ is biholomorphic, that is *f* is holomorphic with its inverse. This is still true if $(\Sigma , g)$, as well as $(\Sigma ', g')$, is a generic 2-dimensional oriented Riemannian smooth manifold, therefore, conformal Riemannian metrics determine the same complex structure in 2-dimensional oriented smooth manifolds, where we recall that two Riemannian metrics $h,h'$ on a same smooth manifold *M* are conformal if there exists $\lambda \in C^\infty (M)$ such that $h'=e^{2\lambda }h$. More precisely, in the case of 2-dimensional oriented smooth manifolds, the concepts of structure complex and of conformal structure (that is a conformal equivalence class of Riemannian metrics) are equivalent.

Above we introduced the notion of a Riemann surface and that a of Riemann surface induced by a 2-dimensional Riemmanian oriented smooth manifold, because we want to apply a known corollary of the Uniformization Theorem, which provides a classification up to biholomorphic maps of all connected Riemann surfaces, to the Riemann surfaces induced by closed connected embedded smooth surfaces of $\mathbb {R}^{3}$ with genus 0. This corollary states that every closed connected Riemann surface of genus zero is biholomorphic equivalent to the Riemann sphere, that is $\mathbb {S}^2\subseteq \mathbb {R}^3$ endowed with the complex structure induced by the Riemmanian oriented surface $(\mathbb {S}^2, g_{\mathbb {S}^2}=\vec {\iota }^{\,\,*}g_{\mathbb {R}^3})$ whose orientation is given once again through (2.5). Here, the expression “biholomorphic equivalent” means that there exists a biholomorphic map between the considered Riemann surfaces. We refer the reader to [21, 22] for a detailed treatment of these topics.

Arguing as in the proof [34, Proposition 3.1]andusingboththeabovecitedcorollaryand [60, Theorem5.1], we obtain the following useful result:

#### Proposition 2.1

For every $\delta _1 \in (0,8\pi )$, there exists a positive constant $C =C(\delta _1)$ with the following property. Let $\Sigma \subset \mathbb {R}^3$ be a closed connected embedded smooth surface of genus 0 with ${\mathcal {H}}^2(\Sigma ) = 4 \pi$ and

$$\begin{aligned} \Vert \mathrm {\text{\AA }}_\Sigma \Vert _{L^2(\Sigma )}^2 < 8\pi - \delta _1. \end{aligned}$$

The Riemmanian surface $\Sigma$ is oriented with orientation given through (2.5). Then there is a smooth conformal orientation-preserving diffeomorphism $f: \mathbb {S}^2 \rightarrow \Sigma$ with pull-back metric $g = f^* g_\Sigma = e^{2u} g_{\mathbb {S}^2}$ having

$$\begin{aligned} \Vert u\Vert _{L^\infty (\mathbb {S}^2)} \le C. \end{aligned}$$

In the that follows we will also need the Michael-Simon inequality, [45], that states the existence of constant $C>0$ such that

2.13 $$\begin{aligned} \Vert w\Vert _{L^{2}(\Sigma )} \le C \int _{\Sigma } |\nabla w| + |\textrm{H}_\Sigma w|\, d {\mathcal {H}}^2 \end{aligned}$$

for every $w \in C^1(\Sigma )$ and for all closed connected embedded smooth surfaces $\Sigma$ of $\mathbb {R}^3$.

### Weak Immersions of the Sphere $\mathbb {S}^2$, Convergence Results and Spheres Minimizing the Canham–Helfrich Energy

Let us consider the Riemannian oriented surface $(\mathbb {S}^2, g_{\mathbb {S}^2})$ with orientation given through (2.5). The default metric on the sphere $\mathbb {S}^2$ will always be the canonical one $g_{\mathbb {S}^2}$, therefore, if not mentioned otherwise, the quantities considered on the sphere $\mathbb {S}^2$ all refer to the standard metric $g_{\mathbb {S}^2}$.

A map $\vec {f}\in W^{2,2}(\mathbb {S}^2,\mathbb {R}^3)\cap W^{1,\infty }(\mathbb {S}^2,\mathbb {R}^3)$ is called a *weak immersion* if the following conditions are satisfied:

- (*i*) There exists $C\ge 1$ such that
  $$\begin{aligned} C^{-1}g_{\mathbb {S}^2}(p)(v,v)\le g(p)(v,v)\le C g_{\mathbb {S}^2}(p)(v,v), \end{aligned}$$
  for every $v\in T_p\mathbb {S}^2$ and almost every $p\in \mathbb {S}^2$, where *g* is the $L^\infty$-metric on $\mathbb {S}^2$ associated with $\vec {f}$ given almost everywhere by $g(\partial _{x^i},\partial _{x^j})=\partial _{x^i}\vec {f} \cdot \,\partial _{x^j}\vec {f}$ in local coordinates. Similarly to the smooth case, the metric *g* induces a Radon measure $\sigma _g$ on $\mathbb {S}^2$, called area measure.
- (*ii*) The Gauss map *N*, or $N_{\vec {f}}$ , defined almost everywhere in any positive local chart of $\mathbb {S}^2$ through the formula (2.6), belongs to $W^{1,2}(\mathbb {S}^2,\mathbb {R}^3)$. Observe that $N\in W^{1,2}(\mathbb {S}^2,\mathbb {R}^3)$ if
  $$\begin{aligned} \int _{\mathbb {S}^2}\!|dN|_g^2\, d\sigma _g<+\infty , \end{aligned}$$
  where $|dN|_g^2=g^{i k}g^{jl} (\partial _{x^{i}}N\cdot \partial _{x^{j}}\vec {f})\,(\partial _{x^{k}}N\cdot \partial _{x^{l}}\vec {f})$ in local coordinates.

We denote by $\mathcal {E}$ the class of the weak immersions $\vec {f}:\mathbb {S}^2\rightarrow \mathbb {R}^3$ of $\mathbb {S}^2$ into $\mathbb {R}^3$.

A map $\vec {f}\in W^{1,\infty }(\mathbb {S}^2,\mathbb {R}^3)$ is *(weakly) conformal* if *g* defined as before can be written almost everywhere as $e^{2u}g_{\mathbb {S}^2}$ for some $u\in L^\infty (\mathbb {S}^2)$ called conformal factor. In this case, the equalities $\partial _{x^1}\vec {f}\cdot \partial _{x^2}\vec {f}=0$ and $|\partial _{x^1}\vec {f}|=|\partial _{x^2}\vec {f}|$ hold almost everywhere for every conformal local chart of $\mathbb {S}^2$. Finally, we recall that for every weak immersion $\vec {f}:\mathbb {S}^2\rightarrow \mathbb {R}^3$, there always exists a bilipschitz homeomorphism $\phi$ of $\mathbb {S}^2$ such that $\vec {f}\circ \phi$ belongs to $W^{2,2}(\mathbb {S}^2,\mathbb {R}^3)\cap W^{1,\infty }(\mathbb {S}^2,\mathbb {R}^3)$, $\vec {f}\circ \phi$ is weakly conformal and $N_{\vec {f}}\circ \phi =N_{\vec {f}\,\circ \, \phi }$, [18, 56].

Given a weak immersion $\vec {f}:\mathbb {S}^2\rightarrow \mathbb {R}^3$, one may define the second fundamental form $\textrm{A}$, the (scalar) mean curvature $\textrm{H}$, the vector mean curvature $\vec {\textrm{H}}$ and the Gaussian curvature $\textrm{K}^G$ almost everywhere in the same way as with smooth immersions.

#### Proposition 2.2

( [56, pp. 81-83]) Let $\vec {f}_1, \vec {f}_2, \dots \in \mathcal {E}$ be a sequence of conformal weak immersions of the 2-sphere $\mathbb {S}^2$ into $\mathbb {R}^3$. We assume that there exist a radius *R* and constant $C>1$ such that

$$\begin{aligned} \vec {f}_k(\mathbb {S}^2)\subset B_R(0)\quad \quad \quad \quad \Vert u_k\Vert _{L^{\infty }(\mathbb {S}^2)}\le C\quad \quad \quad \quad \int _{\mathbb {S}^2} \textrm{H}_{k}^2\,d\sigma _{k}\le C \end{aligned}$$

for all $k\in \mathbb {N}$, where the $u_k$’s, the $\sigma _k$’s and the $\textrm{H}_{k}$’s are the conformal factors, the area measures and the mean curvatures corresponding to the $\vec {f}_{k}$’s, respectively. Then, by possibly passing to a sub-sequence, there exists a map $\vec {f}_{\infty }\in W^{2,2}(\mathbb {S}^2,\mathbb {R}^3)\cap W^{1,\infty }(\mathbb {S}^2,\mathbb {R}^3)$ such that it is weakly conformal and

2.14 $$\begin{aligned} \log (|\nabla \vec {f}_{k}|)&\overset{*}{\rightharpoonup }\ \log (|\nabla \vec {f}_{\infty }|) \quad \text {in}\quad L^{\infty }(\mathbb {S}^2) \end{aligned}$$

2.15 $$\begin{aligned} \vec {f}_k&\rightharpoonup \vec {f}_\infty \quad \quad \quad \quad \text {in}\quad W^{2,2}(\mathbb {S}^2,\mathbb {R}^3) \end{aligned}$$

hold.

We are interested in functionals involving the mean curvature, the area and the (algebraic) volume; Therefore we need to study their behavior under the convergence given by (2.14) and (2.15).

#### Proposition 2.3

( [56, Lemma 5.2] and [47, Lemma 3.1]) Let $\vec {f}_1, \vec {f}_2, \dots \in \mathcal {E}$ be a sequence of conformal weak immersions of the 2-sphere $\mathbb {S}^2$ into $\mathbb {R}^3$, $\sigma _1,\sigma _2,\dots$ the corresponding area measures on $\mathbb {S}^2$, and $N_1, N_2, \dots$ the corresponding Gauss maps. We assume that

$$\begin{aligned} \sup _{k\in \mathbb {N}} \int _{\mathbb {S}^2} |d N_k|_{g_k}^2d\sigma _{g_k}<+\infty \end{aligned}$$

and there exists a weakly conformal map $\vec {f}_{\infty }\in W^{2,2}(\mathbb {S}^2, \mathbb {R}^3)\cap W^{1,\infty }(\mathbb {S}^2, \mathbb {R}^3)$ such that (2.14) and (2.15) hold. Then, $\vec {f}_{\infty }\in \mathcal {E}$, i.e., it is a weak immersion and, by possibly passing to a sub-sequence, it holds that

2.16 $$\begin{aligned} \lim _{k\rightarrow +\infty }\int _{\mathbb {S}^2}d\sigma _{k}&= \int _{\mathbb {S}^2}d\sigma _\infty \end{aligned}$$

2.17 $$\begin{aligned} \lim _{k\rightarrow +\infty }\int _{\mathbb {S}^2} N_{k}\cdot \vec {f}_{k}\,d\sigma _{k}&= \int _{\mathbb {S}^2} N_{\infty }\cdot \vec {f}_{\infty }\,d\sigma _\infty \end{aligned}$$

2.18 $$\begin{aligned} \lim _{k\rightarrow +\infty }\int _{\mathbb {S}^2} \textrm{H}_{k}\,d\sigma _{k}&= \int _{\mathbb {S}^2} \textrm{H}_\infty \,d\sigma _\infty \, \end{aligned}$$

2.19 $$\begin{aligned} \int _{\mathbb {S}^2}\textrm{H}^2_\infty \,d\sigma _\infty&\le \liminf _{k\rightarrow +\infty }\int _{\mathbb {S}^2} \textrm{H}_{k}^2\,d\sigma _{k}, \end{aligned}$$

2.20 $$\begin{aligned} \int _{\mathbb {S}^2}\vert dN_{\infty } \vert ^2_{g_{\infty }}\,d\sigma _{\infty }&\le \liminf _{k\rightarrow +\infty }\int _{\mathbb {S}^2}\vert dN_{k}\vert ^2_{g_k} \,d\sigma _k.. \end{aligned}$$

Another ingredient for the proof of Theorem 1.1 is a compactness result for weak immersions of the 2-sphere. In order to state it, we need to introduce some definitions. For any weak immersion $\vec {f}\in \mathcal {E}$, we define *Canham–Helfrich energy* as

2.21 $$\begin{aligned} \mathcal {H}_{c_0}(\vec {f})=\frac{1}{4}\int _{\mathbb {S}^2}(\textrm{H}-c_0)^2\,d\sigma _{g}, \end{aligned}$$

where $c_0$ is a given real constant. Observe that if $c_0=0$, the Canham–Helfrich energy of $\vec {f}$ coincides with the Willmore energy $\mathcal {W}(\vec {f})$, which is defined by the formula (2.11), exactly as with smooth immersions. Finally, we define by

2.22 $$\begin{aligned} \mathcal {A}(\vec {f})=\int _{\mathbb {S}^2}d\sigma _{g} \quad \quad \text {and}\quad \quad \mathcal {V}(\vec {f})=-\frac{1}{3}\int _{\mathbb {S}^2} N \cdot \vec {f}\,d\sigma _{g} \end{aligned}$$

the area and the (algebraic) volume of $\vec {f}$. In the case that $\vec {f}$ is a smooth embedding and *N* is the inner unit normal, $\mathcal {V}(\vec {f})$ coincides with the enclosed volume, by the divergence theorem (see [59] for instance). The previously mentioned compactness result is.

#### Theorem 2.4

[47] Let $c_0\in \mathbb {R}$ and let $A_0, V_0$ be positive real numbers which satisfy the isoperimetric inequality $36\pi V_0^2\le A_0^3$. Consider the problem 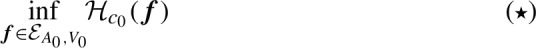 where $\mathcal {E}_{A_0, V_0}=\{\vec {f}\in \mathcal {E}:\,\mathcal {A}(\vec {f})=A_0\,\,\text {and}\,\,\mathcal {V}(\vec {f})=V_0\}$. If there exists a minimizing sequence $\{\vec {f}_k\}_{k\in \mathbb {N}}$ of the problem ($\star$) having

2.23 $$\begin{aligned} \sup _{k\in \mathbb {N}}\mathcal {W}(\vec {f}_k)<8\pi , \end{aligned}$$

then the infimum is attained by a smooth embedding $\vec {f}:\mathbb {S}^2\rightarrow \mathbb {R}^3$.

One may obtain Theorem 2.4 from [47, Theorem 1.7] since the assumption (2.23) on the low Willmore energy prevents the bubbling phenomenon and the presence of branch points. However, for the reader’s convenience we give here a more direct argument, but using the results from [46, 47, 56], that leads to Theorem 2.4.

#### Proof

First of all, note that without loss of generality, we may assume that each $\vec {f}_k$ is also weakly conformal (otherwise, one may replace $\vec {f}_k$ with $\vec {f}_k\circ \phi _k$ for an appropriate bilipschitz homeomorphism $\phi _k$ of $\mathbb {S}^2$, see above). Then, for every $k\in \mathbb {N}$, we have by the Gauss–Bonnet theorem for conformal weak immersion that

2.24 $$\begin{aligned} \int _{\mathbb {S}^2}\vert d N_{k}\vert _{g_{k}}^2 \,d\sigma _{k}\,\le \,4\mathcal {W}(\vec {f}_k)\,\le \,32\pi . \end{aligned}$$

We note that the Gauss–Bonnet theorem for conformal weak immersions is a consequence of the extension of Liouville’s equation to the conformal weak immersions, [56, pp. 96]. Moreover, arguing as in [61, Lemma 1.1] we have the inequalities

2.25 $$\begin{aligned} \sqrt{A_0/(8\pi )}\,&\le \,\sqrt{\mathcal {A}(\vec {f}_k)/\mathcal {W}(\vec {f}_k)}\,\le \,\, \text {diam}(\vec {f}_k(\mathbb {S}^2))\,\nonumber \\&\le \,C\sqrt{\mathcal {A}(\vec {f}_k)\,\mathcal {W}(\vec {f}_k)}\,\le \,C\sqrt{8\pi A_0} \end{aligned}$$

for some constant $C>0$ independent of *k*. Indeed for every $\vec {f}\in \mathcal {E}$, the push forward measure $\mu =\vec {f}_{*}(\sigma _g)$ defines a 2-dimensional integral varifold in $\mathbb {R}^3$, given by the pair $(\vec {f}(\mathbb {S}^2), \sharp (\vec {f}^{-1}(x)))$, with square integrable generalized mean curvature $\textrm{H}_{\mu }$, defined almost everywhere as

$$\begin{aligned} \textrm{H}_{\mu }(x)= \frac{1}{\sharp (\vec {f}^{-1}(x))}\sum _{y\in \vec {f}^{-1}(x)}\!\!\textrm{H}_{\vec {f}}\,(y)\quad \quad \text {if} \,\,\,\sharp (\vec {f}^{-1}(x))>0 \end{aligned}$$

and 0 otherwise. Another consequence of this fact is that

2.26 $$\begin{aligned} \mathcal {W}(\vec {f})\ge 4\pi \lim _{\rho \rightarrow 0^+} \frac{\mu (B_{\rho }(x))}{\pi \rho ^2} \end{aligned}$$

for every $x \in \vec {f}(\mathbb {S}^2)$, which in turn yields $\mathcal {W}(\vec {f})\ge 4\pi$. We will refer to the above limit as density of $\vec {f}$ at *x*. (See [31, Section 2.2] for this last part.)

Applying then [46, Lemma 4.1] with a diagonal argument, we obtain, by possibly passing to a sub-sequence, that for every *k* there exists a smooth conformal orientation-preserving diffeomorphism $\phi _k$ of $\mathbb {S}^2$ such that, for the reparametrized immersion $\vec {F}_k=\vec {f}_k\circ \phi _k\in \mathcal {E}$ and for the new conformal factor $\lambda _k=\frac{1}{2}\log \big (\frac{1}{2}|\nabla \vec {F}_k|^2 \big )$ the following holds: there exists a finite set of points $\{a_1, \dots , a_N\}\subset \mathbb {S}^2$ such that for any compact subset *K* of $\mathbb {S}^2\setminus \{a_1, \dots , a_N\}$ there exists a constant $C_K$ depending on the compact *K* and on the bounds of the areas and energies of the $\vec {f}_k$’s such that $\Vert \lambda _k\Vert _{L^{\infty }(K)}\le ~C_K.$ Moreover, since the diameters of the images of our immersions are bounded uniformly, see (2.25), then by possibly translating the sets we may assume that the $\vec {F}_k(\mathbb {S}^2)$’s are contained in the ball $B_R(0)$. Then, in any compact subset of $\mathbb {S}^2\setminus \{a_1, \dots , a_N\}$ the assumptions of Proposition 2.2 are satisfied, therefore using the argument from [56, pp. 81-83] together with a standard diagonal argument we deduce that there exists a map $\vec {F}_\infty :\mathbb {S}^2\setminus \{a_1, \dots , a_N\}\rightarrow \mathbb {R}^3$ such that $\vec {F}_\infty \in W^{2,2}_{loc}(\mathbb {S}^2\setminus \{a_1, \dots , a_N\},\mathbb {R}^3)\cap W^{1,\infty }_{loc}(\mathbb {S}^2\setminus \{a_1, \dots , a_N\},\mathbb {R}^3)$, $\vec {F}_\infty$ is weakly conformal,

$$\begin{aligned} \log (|\nabla \vec {F}_k|)&\overset{*}{\rightharpoonup }\ \log (|\nabla \vec {F}_\infty |) \quad \text {in}\quad L^{\infty }_{loc}(\mathbb {S}^2\setminus \{a_1, \dots , a_N\})\\ \vec {F}_k&\rightharpoonup \vec {F}_\infty \quad \quad \quad \quad \,\,\,\text {in}\quad W^{2,2}_{loc}(\mathbb {S}^2\setminus \{a_1, \dots , a_N\},\mathbb {R}^3). \end{aligned}$$

Moreover, the equalities and inequalities of Proposition 2.3, i.e. (2.16)–(2.20), are true replacing $\mathbb {S}^2$ with $\mathbb {S}^2\setminus \cup _{j=1}^N B_{\varepsilon }(a_j)$, for any $\varepsilon >0$. Joining all this information with the fact that the $\vec {F}_k$’s have the same area and are contained in the same ball, one may also deduce that $\vec {F}_\infty$ extends to a (weakly conformal) map in $W^{1,2}(\mathbb {S}^2,\mathbb {R}^3)$, as well as $N_{\infty }$ and $e^{2\lambda _\infty }\textrm{H}^2_{\infty }$ extend to a map in $W^{1,2}(\mathbb {S}^2,\mathbb {R}^3)$ and in $L^{2}(\mathbb {S}^2)$ respectively, and that

$$\begin{aligned} \vec {F}_k\overset{*}{\rightharpoonup }\ \vec {F}_\infty \quad \text {in}\quad L^{\infty }(\mathbb {S}^2)\quad \quad \text {and} \quad \quad \vec {F}_k\rightharpoonup \vec {F}_\infty \quad \text {in}\quad W^{1,2}(\mathbb {S}^2,\mathbb {R}^3); \end{aligned}$$

see [56, pp. 89-90]. From [56, Lemma 6.2] and [28, Section 4.2.3] it follows then that $\vec {F}_\infty$ belongs to $W^{1,\infty }(\mathbb {S}^2,\mathbb {R}^3)$, which in turn implies that $\vec {F}_\infty \in W^{2,2}(\mathbb {S}^2,\mathbb {R}^3)$, and that for each $a_j$ there exists a positive integer $n_j$ such that the singularity can be removed if $n_j=1$, and the density of $\vec {F}_\infty$ at $\vec {F}_\infty (a_i)$ is always bigger or equal to $n_j$. Therefore, having that

$$\begin{aligned} 4\pi n_j\le \mathcal {W}(\vec {F}_\infty )\le \liminf _{k\rightarrow +\infty }\mathcal {W}(\vec {F}_k)<8\pi , \end{aligned}$$

one obtains that $\vec {F}_\infty \in \mathcal {E}$.

In order to deduce that $\vec {F}_\infty$ is a minimizer of the problem ($\star$), it remains to show that

2.27 $$\begin{aligned} \mathcal {H}_{c_0}(\vec {F}_\infty )\le \inf _{ \vec {f}\in \mathcal {E}_{A_0, V_0}}\mathcal {H}_{c_0}(\vec {f})\,\quad \quad \quad \quad \mathcal {A}(\vec {F}_\infty )=A_0\quad \quad \quad \quad \mathcal {V}(\vec {F}_\infty )=V_0 \end{aligned}$$

In particular, $\vec {F}_\infty$ then satisfies Euler-Lagrange equation associated with the Canham–Helfrich energy in a weak sense and, as a consequence, it is smooth by following the proof of [47, Theorem 4.3].

Notice that in order to have (2.27), it is enough to show that

$$ \lim _{\varepsilon \rightarrow 0^+}\liminf _{k\rightarrow +\infty } \mathcal {A}\big (\vec {F}_k\,|_{B_{\varepsilon }(a_j)}\,\big )\rightarrow 0 $$

for each $a_j$. Again this is a consequence of the fact that $\sup _{k\in \mathbb {N}}\mathcal {W}(\vec {F}_k)<8\pi$. Indeed, we argue by contradiction and assume that there exists $j_0\in \{1,\dots ,N\}$ for which $\lim \limits _{\varepsilon \rightarrow 0^+}\liminf \limits _{k\rightarrow +\infty }\mathcal {A}\big (\vec {F}_k\,|_{B_{\varepsilon }(a_{j_0})}\big )>0$. Then, by [28, Lemma 4.4], $\lim \limits _{\varepsilon \rightarrow 0^+}\liminf \limits _{k\rightarrow +\infty }\mathcal {W}\big (\vec {F}_k\,|_{B_{\varepsilon }(a_{j_0})}\big )\ge 4\pi$. However, this is impossible, as

$$\begin{aligned} 8\pi&> \lim _{\varepsilon \rightarrow 0^+}\liminf _{k\rightarrow +\infty }\Big (\mathcal {W}\big (\vec {F}_k\,|_{\cup _{j=1}^N B_{\varepsilon }(a_{j})}\,\big )+\mathcal {W}\big (\vec {F}_k\,|_{\mathbb {S}^2\setminus \cup _{j=1}^N B_{\varepsilon }(a_{j})}\,\big )\Big )\\&\ge 4\pi +\mathcal {W}(\vec {F}_\infty )\ge 8\pi \,. \end{aligned}$$

Finally, since $\vec {F}_\infty$ is a smooth conformal map, it is, in particular, a smooth immersion. The fact that $\vec {F}_\infty$ is injective, hence a smooth embedding, follows from (2.26) since the density of $\vec {F}_\infty$ at *x*, for any $x\in \vec {F}_\infty (\mathbb {S}^2)$, coincides with $\sharp (\vec {F}_{\infty }^{-1}(x))$, and $\mathcal {W}(\vec {F}_\infty )<8\pi$. $\square$

## Consequences of the Non-sharp quantitative Alexandrov theorem [25]

Let us begin by restating [25, Theorem 1.2] in a slightly different way.

### Theorem 3.1

Let $E \subset \mathbb {R}^3$ be $C^2$-regular set such that $P(E) \le C_0$ and $|E| = |B_1|$. There are constants $q \in (0,1], C>1$ and $\tau >0$, depending only on $C_0$, such that if $\Vert \textrm{H}_E-\bar{\mathrm {{H}}}_E\big \Vert _{L^2(\partial E)}\le \tau$ then $C^{-1} \le \bar{\mathrm {{H}}}_E \le C$ and there are points $x_1, \dots , x_N$ with $|x_i - x_j|\ge 2 \rho$ for $i \ne j$, where $\rho = 2 \bar{\mathrm {{H}}}^{-1}$, such that for $F = \cup _{i=1}^N B_\rho (x_i)$ it holds that

$$ \sup _{x\in E \Delta F} |d_{F}| + \big |P(E) - 4 \pi N^{\frac{1}{3}} \big | \le C \big \Vert \textrm{H}_E-\bar{\mathrm {{H}}}_E\big \Vert _{L^2(\partial E)}^q. $$

Indeed, from [25, Theorem 1.2, Remark 3.1] we know that, if $\Vert H_E-{\bar{H}}_E\big \Vert _{L^2(\partial E)}\le \tau$, then $1/C<{\bar{H}}_E<C$, and that *E* contains a number *N* of balls of radius $r_-=\rho -C\Vert H_E-{\bar{H}}_E\big \Vert _{L^2(\partial E)}^{q}$ and is contained in *N* balls of radius $r_+=\rho +C\Vert H_E-{\bar{H}}_E\big \Vert _{L^2(\partial E)}^{q}$, where the constants $q,\,C,\,\tau ,\,\rho$ are those of Theorem 3.1. Requiring that $\tau \in (0,1)$, then the volume constraint $|E|=|B_1|$ implies that

3.1 $$\begin{aligned} |N- \rho ^{-3}|\le C(C_0)\Vert \textrm{H}_E-\bar{\mathrm {{H}}}_E\Vert _{L^2(\partial E)}^q. \end{aligned}$$

Theorem 3.1 implies the following non-sharp version of Theorem 1.1:

### Corollary 3.2

There exists $\tau _0\in (0,1)$, depending on $\delta _0$ in condition (1.1), such that for every $C^2$-regular set $E \subset \mathbb {R}^3$ which satisfies (1.1) and $\big \Vert \textrm{H}_E-\bar{\mathrm {{H}}}_E\big \Vert _{L^2(\partial E)}\le \tau _0$ it holds that

3.2 $$\begin{aligned} |\bar{\mathrm {{H}}}_E-2| \,+ \sup _{x\in E \Delta B_1(x_0)} \big ||x - x_0|-1\big | + \big |P(E) - P(B_1)\big | \le C \big \Vert \textrm{H}_E-\bar{\mathrm {{H}}}_E\big \Vert _{L^2(\partial E)}^q, \end{aligned}$$

for a suitable $x_0 \in \mathbb {R}^3$, with $q\in (0,1]$ and $C>0$ as in Theorem 3.1.

### Proof

Theorem 3.1 and the condition (1.1) yield that

$$ 4 \pi N^{\frac{1}{3}} \le P(E) + C\tau _0^q \le 4 \pi \root 3 \of {2}+ C\tau _0^q -\delta _0 $$

for a number $N \in \mathbb {N}$. When $\tau _0$ is small enough (depending on $\delta _0$), the above implies $N < 2$ and thus $N= 1$. The inequality (3.1) implies the rest of the statement. $\square$

In turn, Corollary 3.2 allows us to establish the following powerful result, which contains information on the topology of the sets:

### Corollary 3.3

There exists $\tau _0\in (0,1)$, depending on $\delta _0$ in condition (1.1), such that, for every $C^\infty$-regular set $E \subset \mathbb {R}^3$ which satisfies (1.1) and $\big \Vert \textrm{H}_E-\bar{\mathrm {{H}}}_E\big \Vert _{L^2(\partial E)}\le \tau _0$, the boundary $\partial E$ of *E* is a closed connected embedded smooth surface of $\mathbb {R}^3$ with genus 0, which admits a smooth conformal orientation-preserving diffeomorphism $f: \mathbb {S}^2 \rightarrow \partial E$ and the conformal factor $u\in C^{\infty }(\mathbb {S}^2)$, defined by $f^* g_{\partial E} = e^{2u} g_{\mathbb {S}^2}$, satisfies

3.3 $$\begin{aligned} \Vert u\Vert _{L^\infty (\mathbb {S}^2)} \le C, \end{aligned}$$

where $C>0$ is a constant depending on $\delta _0$. Here, the fixed orientation of $\partial E$ is that one given through (2.5).

Observe that *E* is connected as a consequence of the connectedness of $\partial E$.

### Proof

By Corollary 3.2, we have for $\rho =2\textrm{H}_E^{-1},$ that

3.4 $$\begin{aligned} \int _{\partial E} \textrm{H}_E^2 \, d{\mathcal {H}}^2 - 16 \pi&=\int _{\partial E} \textrm{H}_E^2 \, d{\mathcal {H}}^2 - \bar{\mathrm {{H}}}_E^2\, P(E) + \bar{\mathrm {{H}}}_E^2\,\big ( P(E)-P(B_1)\big )\nonumber \\&\quad +4\pi \,\bar{\mathrm {{H}}}_E^2\,\big (1-\rho ^2\big ) \le C\, \big \Vert \textrm{H}_E-\bar{\mathrm {{H}}}_E\big \Vert _{L^2(\partial E)}^q\le C\,\tau _0^q. \end{aligned}$$

Choosing to be $\tau _0>0$ small enough, the boundary $\partial E$ of *E* is connected and of genus zero due to the properties of the Willmore functional discussed at the end of Subsection 2.1. Now we use Gauss-Bonnet theorem (2.12), the fact that the genus of $\partial E$ is zero and the estimate (3.4) in order to write that

3.5 $$\begin{aligned} \int _{\partial E} |\mathrm {\text{\AA }}_{E}|^2\, d\mathcal {H}^2&=\frac{1}{2}\int _{\partial E} \textrm{H}_{E}^2\, d\mathcal {H}^2-2\int _{\partial E} \textrm{K}^G_{E}\, d\mathcal {H}^2 =\frac{1}{2}\int _{\partial E} \textrm{H}_{E}^2\, d\mathcal {H}^2- 4\pi \chi (\partial E)\nonumber \\&\le C\, \big \Vert \textrm{H}_E-\bar{\mathrm {{H}}}_E\big \Vert _{L^2(\partial E)}^q \le C\tau _0^{q}. \end{aligned}$$

Recall that $\mathrm {\text{\AA }}_{E}$ denotes the traceless part of the second fundamental form and $\textrm{K}^G_{E}$ the Gaussian curvature of $\partial E$, defined in Subsection 2.1. Therefore, by decreasing $\tau _0>0$ if necessary, we may assume that $\Vert \mathrm {\text{\AA }}_{E}\Vert _{L^2(\partial E)}^2<2\pi$. This allows us to apply Proposition 2.1 to the set $F=\alpha E$ such that $P(F)=4\pi$, as the dilatation invariance yields $\int _{\partial F} |\mathrm {\text{\AA }}_{F}|^2\, d\mathcal {H}^2=\int _{\partial E} |\mathrm {\text{\AA }}_{E}|^2\, d\mathcal {H}^2\,$. Hence, there exist a universal constant ${\bar{C}}>0$ and a smooth conformal orientation-preserving diffeomorphism $f:\mathbb {S}^2 \rightarrow \partial E$ such that

3.6 $$\begin{aligned} \Vert u+\log \alpha \Vert _{L^\infty (\mathbb {S}^2 )}\le {\bar{C}}, \end{aligned}$$

where $f^*g_{\partial E}=e^{2u} g_{\mathbb {S}^2}$. We obtain the estimate (3.3) from the conditions in (1.1). $\square$

## Proof of Theorem 1.1

We will prove Theorem 1.1 with a variational argument by converting the inequality into a minimization problem. The core of the paper is proving the following result, which then implies Theorem 1.1 by a rather straightforward argument:

### Proposition 4.1

There exists ${\bar{\varepsilon }}={\bar{\varepsilon }}(\delta _0)\in (0,1)$ such that for all $\varepsilon \in (0, {\bar{\varepsilon }})$ the functional 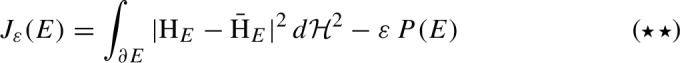 is minimized by the unit ball $B_1$ in the class of $C^2$-regular sets $E \subset \mathbb {R}^3$ which satisfy (1.1), i.e., $|E|=|B_1|$ and $P(E)\le 4 \pi \root 3 \of {2} -\delta _0$.

### Proof

Let us divide the proof into two steps.

**Step 1:** *There exists *${\bar{\varepsilon }}\in (0,1)$* such that for all *$\varepsilon \in (0, {\bar{\varepsilon }})$*, the functional *$J_{\varepsilon }$* admits a smooth minimizer *$F=F(\varepsilon )$* in the class of the considered sets.* We begin by choosing a minimizing sequence $(E_n)_n$ of $C^2$-regular sets which satisfy (1.1) and converges to the infimum value of $J_\varepsilon$. By an approximation argument, we may assume that the minimizing sequence $(E_n)_n$ is smooth, i.e., $\partial E_n$ are $C^\infty$-surfaces. We may also assume small oscillation of the mean curvature:

4.1 $$\begin{aligned} \big \Vert \textrm{H}_{E_n}- {\bar{\mathrm {{H}}}}_{E_n}\big \Vert _{L^2(\partial E_n)}^2 \le 8 \pi {\bar{\varepsilon }}. \end{aligned}$$

Indeed, we may always assume the minimizing sequence to have smaller energy than the ball, i.e., $J_\varepsilon (E_n)\le J_\varepsilon (B_1)$. Therefore, by the perimeter bound in (1.1), it holds that

$$\begin{aligned} \int _{\partial E_n} |\textrm{H}_{E_n}- {\bar{\mathrm {{H}}}}_{E_n}|^2 \, d{\mathcal {H}}^2 \le \varepsilon \left( P(E_n) - P(B_1)\right) \le 4 \pi \root 3 \of {2} \varepsilon \le 8 \pi {\bar{\varepsilon }}. \end{aligned}$$

As important consequences of (4.1) when ${\bar{\varepsilon }}$ is small enough, we have by Corollary 3.2 and its consequences (3.4)-(3.5) that

4.2 $$\begin{aligned} B_{1-C{\bar{\varepsilon }}^{q/2}}(x_n) \subset E_n\subset B_{1+C{\bar{\varepsilon }}^{q/2}}&(x_n) \end{aligned}$$

4.3 $$\begin{aligned} | \bar{\mathrm {{H}}}_{E_n}-2|\le&\, C {\bar{\varepsilon }}^{\frac{q}{2}} \end{aligned}$$

4.4 $$\begin{aligned} 0 \le P(E_n) - P(B_1) \le&\,C {\bar{\varepsilon }}^{\frac{q}{2}} \end{aligned}$$

4.5 $$\begin{aligned} \int _{\partial E_n} \textrm{H}_{E_n}^2 \, d{\mathcal {H}}^2 - 16 \pi \le&\,C {\bar{\varepsilon }}^{\frac{q}{2}} \end{aligned}$$

4.6 $$\begin{aligned} \int _{\partial E_n} |\mathrm {\text{\AA }}_{E_n}|^2\, d\mathcal {H}^2\le&\,C {\bar{\varepsilon }}^{\frac{q}{2}}. \end{aligned}$$

Since the problem is translation invariant, we may assume that each $x_n$ is the origin. Furthermore, again for ${\bar{\varepsilon }}$ small enough, Corollary 3.3 guarantees the existence of a uniform constant *C* and a smooth conformal orientation-preserving diffeomorphism $\vec {f}_n: \mathbb {S}^2 \rightarrow \partial E_n\subset \mathbb {R}^3$ such that

4.7 $$\begin{aligned} \Vert u_n\Vert _{L^\infty (\mathbb {S}^2)} \le C, \end{aligned}$$

where $u_n\in C^{\infty }(\mathbb {S}^2)$ is given by $\vec {f}_{n}^{*} g_{\partial E_n}= e^{2u_n} g_{\mathbb {S}^2}$. We recall that as usual, the fixed orientation of $\partial E_n$ is that one given through (2.5).

At this point, we observe that by the above results we may write the energy $J_\varepsilon (E_n)$ using the parametric approach constructed in Subsection 2.5 and have

4.8 $$\begin{aligned} J_\varepsilon (E_n)=\int _{\mathbb {S}^2} (\textrm{H}_{\vec {f_n}}-\bar{\mathrm {{H}}}_{\vec {f}_n})^2\,d\sigma _{g_{\vec {f}_n}}\!\!-\varepsilon \mathcal {A}( \vec {f}_n)=:J_\varepsilon (\vec {f}_n) \end{aligned}$$

and $\mathcal {V}(\vec {f}_n)=|B_1|$, where $\mathcal {A}(\cdot )$ and $\mathcal {V}(\cdot )$ are the area and the (algebraic) volume, defined in (2.22). These equalities follow from the fact that given any positive chart $(U, \varphi )$ of $\mathbb {S}^2$, then $(\vec {f}_n(U), \varphi \circ \vec {f}_n^{-1})$ is a positive chart of $\partial E_n$. Indeed, in such local charts, one can check that

$$\begin{aligned} \partial _{x^i}^{\partial E_n}|_q&=\partial _{x^i}\vec {f}_n|_{\vec {f}_n^{-1}(q)}g^{\partial E_n}_{ij}(q)= g^{\vec {f}_n}_{ij}(\vec {f}_n^{-1}(q))\sqrt{\det (g^{\partial E_n}_{ij})(q)}\\&=\sqrt{\det ( g^{\vec {f}_n}_{ij})(\vec {f}_n^{-1}(q))}\,N^{\partial E_n}(q)=N^{\vec {f}_n}(\vec {f}_n^{-1}(q))\,\, \textrm{A}^{\partial E_n}_{ij}(q)\\&=\textrm{A}^{\vec {f}_n}_{ij}(\vec {f}_n^{-1}(q))\,\textrm{H}_{\partial E_n}(q)=\textrm{H}_{\vec {f}_n}(\vec {f}_n^{-1}(q)). \end{aligned}$$

Joining all of the above, one gets the equality (4.8), while the identity $\mathcal {V}(\vec {f}_n)=|B_1|$ follows by also applying the divergence theorem.

Therefore the sequence of the smooth conformal immersions $\vec {f}_n$ of the 2-sphere $\mathbb {S}^2$ into $\mathbb {R}^3$ satisfy the assumptions of Proposition 2.2 and by (2.24) also the assumptions of Proposition 2.3. Thus, by possibly passing to a converging sub-sequence, we deduce that at the limit there exists a conformal weak immersion $\vec {f}_{\infty }\in \mathcal {E}$ of the 2-sphere $\mathbb {S}^2$ into $\mathbb {R}^3$ such that the equalities and inequalities (2.16)–(2.20) are true.

Using the notation from Section 2.3, we then have

$$\begin{aligned} \inf _{\begin{array}{c} E\subset \mathbb {R}^3 \\ \text {satisfies }(1.1) \end{array}}\!\!\!\!\! J_{\varepsilon }(E)\,&=\!\lim _{n\rightarrow +\infty } J_{\varepsilon }(E_{n})=\!\lim _{n\rightarrow +\infty } J_{\varepsilon }(\vec {f}_{n})\,\ge \, J_{\varepsilon }(\vec {f}_\infty )\\&=\!\int _{\mathbb {S}^2} (\textrm{H}_\infty -\bar{\mathrm {{H}}}_\infty )^2\,d\sigma _{\infty } -\varepsilon \mathcal {A}(\vec {f}_\infty ) \end{aligned}$$

and $\mathcal {V}(\vec {f}_\infty )=|B_1|$. The map $\vec {f}_{\infty }\in \mathcal {E}$ is then the bridge between the two variational problems by virtue of the crucial chain of inequalities

4.9 $$\begin{aligned} \inf _{\begin{array}{c} E\subset \mathbb {R}^3 \\ \text {satisfies }(1.1) \end{array}}\!\!\!\!\! J_{\varepsilon }(E)\,\ge \,J_{\varepsilon }(\vec {f}_\infty ) \,\ge \, 4 \bigg (\inf _{\vec {h}\in \mathcal {E}_{A_\infty ,V_\infty }}\!\!\!\mathcal {H}_{\bar{\mathrm {{H}}}_\infty }(\vec {h})\,\bigg ) -\varepsilon A_\infty , \end{aligned}$$

where $A_\infty =\mathcal {A}(\vec {f}_\infty )$, $V_\infty = \mathcal {V}(\vec {f}_\infty )$ and $\mathcal {E}_{A_\infty , V_\infty }=\{\vec {f}\in \mathcal {E}:\,\mathcal {A}(\vec {f})=A_\infty \,\,\text {and}\,\,\mathcal {V}(\vec {f})=V_\infty \}$. Here, $\mathcal {H}_{\bar{\mathrm {{H}}}_\infty }(\vec {h})$ is the Canham–Helfrich energy of the weak immersion $\vec {h}\in \mathcal {E}$, defined in (2.21) with $c_0 = \bar{\mathrm {{H}}}_\infty$. Therefore, next we show that the assumptions of Proposition 2.4 are satisfied.

We begin by observing that joining the limit information (2.16)–(2.19) with the inequalities (4.1), (4.3), (4.4) we know that the estimates

4.10 $$\begin{aligned} 0\le A_\infty \!-4\pi \le C {\bar{\varepsilon }}^{\frac{q}{2}}\quad \quad \text {and} \quad \quad | \bar{\mathrm {{H}}}_{\infty }-2|\le \, C {\bar{\varepsilon }}^{\frac{q}{2}} \end{aligned}$$

hold with the same uniform constant $C>0$ of (4.3)-(4.4), and that

4.11 $$\begin{aligned} \int _{\mathbb {S}^2}(\textrm{H}_{\infty }- {\bar{\mathrm {{H}}}}_{\infty })^2\,d\sigma _{\infty }\le 8 \pi {\bar{\varepsilon }}. \end{aligned}$$

The first consequence of (4.10) is that the positive constants $A_\infty , V_\infty$ satisfy the isoperimetric inequality $36\pi V_\infty ^2\le A_\infty ^3$, as $\mathcal {V}_\infty =|B_1|$. Let $\vec {h}_1, \vec {h}_2, \dots \in \mathcal {E}$ be a minimizing sequence for the problem $\inf _{\vec {h}\in \mathcal {E}_{A_\infty , V_\infty }}\!\!\!\mathcal {H}_{\bar{\mathrm {{H}}}_\infty }(\vec {h})$, $\widetilde{\sigma }_1,\widetilde{\sigma }_2,\dots$ the corresponding area measures, and $\widetilde{\textrm{H}}_1, \widetilde{\textrm{H}}_2, \dots$ the associated mean curvatures. By the Minkowski inequality,

$$\begin{aligned} \Vert \widetilde{\textrm{H}}_n\Vert _{L^2(\mathbb {S}^2,\,\widetilde{\sigma }_n)}&\le \Vert \widetilde{\textrm{H}}_n-\bar{\mathrm {{H}}}_\infty \Vert _{L^2(\mathbb {S}^2,\, \widetilde{\sigma }_n)} +\Vert \bar{\mathrm {{H}}}_\infty \Vert _{L^2(\mathbb {S}^2,\, \widetilde{\sigma }_n)} \\&= \Vert \widetilde{\textrm{H}}_n-\bar{\mathrm {{H}}}_\infty \Vert _{L^2(\mathbb {S}^2,\, \widetilde{\sigma }_n)} +\bar{\mathrm {{H}}}_\infty \sqrt{A}_\infty . \end{aligned}$$

Since $\vec {f}_\infty \in \mathcal {E}_{A_\infty , V_\infty }$, we may assume that $\mathcal {H}_{\bar{\mathrm {{H}}}_\infty }(\vec {h}_n)\le \mathcal {H}_{\bar{\mathrm {{H}}}_\infty }(\vec {f}_\infty )$ for all $n\in \mathbb {N}$. Therefore by (4.10) and (4.11) it holds that

$$\begin{aligned} \Vert \widetilde{\textrm{H}}_n\Vert _{L^2(\mathbb {S}^2, \,\widetilde{\sigma }_n)}&\le \Vert \widetilde{\textrm{H}}_\infty -\bar{\mathrm {{H}}}_\infty \Vert _{L^2(\mathbb {S}^2,\,\sigma _{\infty })} +\bar{\mathrm {{H}}}_\infty \sqrt{A}_\infty \\&\le \, \sqrt{8 \pi {\bar{\varepsilon }}}+(2+C {\bar{\varepsilon }}^{\frac{q}{2}}) \sqrt{4\pi +C {\bar{\varepsilon }}^{\frac{q}{2}}}. \end{aligned}$$

As a consequence, for ${\bar{\varepsilon }}>0$ sufficiently small, the minimizing sequence of the $\vec {h}_n$’s satisfies the condition (2.23) (recall that $\mathcal {W}(\vec {h}_n)=\Vert \widetilde{\textrm{H}}_n\Vert _{L^2(\mathbb {S}^2, \,\widetilde{\sigma }_n)}^2/4$). Thus, applying Theorem 2.4, the infimum of the problem $\inf _{\vec {h}\in \mathcal {E}_{A_\infty , V_\infty }}\!\!\!\mathcal {H}_{\bar{\mathrm {{H}}}_\infty }(\vec {h})$ is attained by a smooth embedding $\vec {h}:\mathbb {S}^2\rightarrow \mathbb {R}^3$. Therefore, as explained in Subsection 2.1, $\vec {h}(\mathbb {S}^2)$ is the $C^\infty$-boundary of a smooth bounded set $F\subset \mathbb {R}^3$, and it also holds $P(F)=A_\infty$, $|F|=|B_1|$ and

$$\begin{aligned} \mathcal {H}_{\bar{\mathrm {{H}}}_\infty }(\vec {h})=\frac{1}{4}\int _{\partial F} (\textrm{H}_{F}-\bar{\mathrm {{H}}}_\infty )^2\, d\mathcal {H}^2. \end{aligned}$$

The conclusion then follows by observing that

$$\begin{aligned} \inf _{\begin{array}{c} E\subset \mathbb {R}^3 \\ \text {satisfies }(1.1) \end{array}}\!\!\!\!\! J_{\varepsilon }(E)&\,\ge 4 \bigg ( \inf _{\vec {h}\in \mathcal {E}_{A_\infty ,V_\infty }}\!\!\!\mathcal {H}_{\bar{\mathrm {{H}}}_\infty }(\vec {h})\,\bigg ) -\varepsilon A_\infty \\&\,\ge \, \int _{\partial F} (\textrm{H}_{F}-\bar{\mathrm {{H}}}_\infty )^2\, d\mathcal {H}^2-\varepsilon P(F)\\&\,\ge \,\int _{\partial F} (\textrm{H}_{F}-\bar{\mathrm {{H}}}_{F})^2\, d\mathcal {H}^2-\varepsilon P(F)\\&\,\ge \inf _{\begin{array}{c} E\subset \mathbb {R}^3 \\ \text {satisfies }(1.1) \end{array}}\!\!\!\!\!J_{\varepsilon }(E). \end{aligned}$$

Hence, $F$ is a smooth minimizer of the functional $J_{\varepsilon }$ in the class of $C^2$-regular sets $E \subset \mathbb {R}^3$ which satisfy (1.1).

**Step 2:** *For each *$\varepsilon \in (0, {\bar{\varepsilon }})$*, possibly choosing a smaller *${\bar{\varepsilon }}>0$*, every smooth minimizer *$F=F(\varepsilon )$* of the functional *$J_{\varepsilon }$* is the unit ball, i.e. up to a translation it holds *$F= B_1$.

Since $F$ is a smooth minimizer of the functional $J_{\varepsilon }$ it satisfies the associated Euler-Lagrange equation

4.12 $$\begin{aligned} -\Delta \textrm{H}_{F} = \vert \textrm{A}_{F}\vert ^2(\textrm{H}_{F}-{\bar{\textrm{H}}}_{F}) - \frac{1}{2} (\textrm{H}_{F}-{\bar{\textrm{H}}}_{F})^{2\,}\textrm{H}_{F} + \frac{\varepsilon }{2}\,\textrm{H}_{F}+ \lambda \qquad \text {on } \, \partial F\end{aligned}$$

in the classical sense, where $\lambda$ is the Lagrange multiplier due to the volume constraint. The derivation of (4.12) is standard, and we merely refer to [20, Theorem 3.2] for the relevant calculations, and thus we omit it.

Let us divide the rest of the proof in sub–steps. In the following *C* will denote a constant which is independent of $\varepsilon \in (0,{\bar{\varepsilon }})$ and may vary from a line to line. Furthermore, we underline that from now on we will use the classical inequalities (2.9) repeatedly. Step 2.1: It holds that

4.13 $$\begin{aligned} |\lambda |&\le C \end{aligned}$$

4.14 $$\begin{aligned} \Vert \textrm{A}_{F}\Vert _{L^4(\partial F)}^4&\le C \end{aligned}$$

4.15 $$\begin{aligned} \Vert \nabla \textrm{A}_{F} \Vert _{L^2(\partial F)}^2&\le C. \end{aligned}$$

Therefore, the classical inequalities (2.9) imply that

4.16 $$\begin{aligned} \Vert \textrm{H}_{F}\Vert _{L^4(\partial F)}^4&\le C \end{aligned}$$

4.17 $$\begin{aligned} \Vert \nabla \textrm{H}_{F} \Vert _{L^2(\partial F)}^2&\le C. \end{aligned}$$

We begin by noticing that $F$ also satisfies (for same reason of the $E_n$’s) the estimates (4.1) and (4.2)-(4.6). In order to estimate the Lagrange multiplier $\lambda$, we integrate (4.12) over $\partial F$, use $\int _{\partial F} \Delta \textrm{H}_{F}\, d{\mathcal {H}}^2 = 0$ and the Young’s inequality. From the estimates on the perimeter and on the integral average of the mean curvature that it follows that

$$\begin{aligned} |\lambda | P(F) \le C\int _{\partial F}(1+ |\textrm{A}_{F}|^3) \, d{\mathcal {H}}^2\le C\int _{\partial F}(1+ |\textrm{A}_{F}|^4) \, d{\mathcal {H}}^2, \end{aligned}$$

which yields that

4.18 $$\begin{aligned} |\lambda | \le C\big (1+ \Vert A_{F} \Vert _{L^4(\partial F)}^4\big ) \end{aligned}$$

as $P(F)\ge 4\pi$. We continue by multiplying the Euler-Lagrange equation (4.12) by $\textrm{H}_{F}$ and integrating by parts. After estimating the right hand side similarly as before, we then obtain that

4.19 $$\begin{aligned} \Vert \nabla \textrm{H}_{F} \Vert _{L^2(\partial F)}^2 \le C(1+ \Vert \textrm{A}_{F} \Vert _{L^4(\partial F)}^4) + |\lambda |\,P(F) \,{\bar{\textrm{H}}}_{F}\le C(1+ \Vert A_{F} \Vert _{L^4(\partial F)}^4 ), \end{aligned}$$

where the last inequality follows from (4.3) and (4.18). We then recall Simon’s identity (2.10). We multiply (2.10) by $\textrm{A}_{F}$ and integrate by parts to get that

$$ \Vert \nabla \textrm{A}_{F} \Vert _{L^2(\partial F)}^2 \le \Vert \nabla \textrm{H}_{F} \Vert _{L^2(\partial F)}^2 + C \Vert \textrm{A}_{F} \Vert _{L^4(\partial F)}^4. $$

Hence, we have, by the two items above, that

4.20 $$\begin{aligned} \Vert \nabla \textrm{A}_{F} \Vert _{L^2(\partial F)}^2 \le C(1+\Vert \textrm{A}_{F} \Vert _{L^4(\partial F)}^4) \le C\big ( 1+\big \Vert \textrm{A}_{F} -\frac{1}{2}\,\bar{\mathrm {{H}}}_{F}\, g \big \Vert _{L^4(\partial F)}^4\big ). \end{aligned}$$

We proceed by using the Michael-Simon inequality (2.13) with the function $u= | \textrm{A}_{F} -\frac{1}{2}\,\bar{\mathrm {{H}}}_{F}\, g|^2$. We have by Cauchy-Schwarz and by (4.20) that

$$\begin{aligned}&\big \Vert \textrm{A}_{F} -\frac{1}{2}\,\bar{\mathrm {{H}}}_{F}\, g\big \Vert _{4}^2 \le C \int _{\partial F} |\nabla \textrm{A}_{F}| \big |\textrm{A}_{F} -\frac{1}{2}\,\bar{\mathrm {{H}}}_{F}\, g\big | + |\textrm{H}_{F}| \big | \textrm{A}_{F} -\frac{1}{2}\,\bar{\mathrm {{H}}}_{F}\, g\big |^2 \, d {\mathcal {H}}^2 \\&\le C \int _{\partial F} |\nabla \textrm{A}_{F}|\big | \textrm{A}_{F} -\frac{1}{2}\,\bar{\mathrm {{H}}}_{F}\, g\big | + |\textrm{H}_{F}-\bar{\mathrm {{H}}}_{F}| \big |\textrm{A}_{F} -\frac{1}{2}\,\bar{\mathrm {{H}}}_{F}\, g \big |^2 +\bar{\mathrm {{H}}}_{F} \big |\textrm{A}_{F} -\frac{1}{2}\,\bar{\mathrm {{H}}}_{F}\, g \big |^2 \, d {\mathcal {H}}^2 \\&\le C\Big (\Vert \nabla \textrm{A}_{F} \Vert _{2}\, \big \Vert \textrm{A}_{F} -\frac{1}{2}\,\bar{\mathrm {{H}}}_{F}\, g \big \Vert _{2} +\Vert \textrm{H}_{F}-\bar{\mathrm {{H}}}_{F} \Vert _{2}\, \big \Vert \textrm{A}_{F} -\frac{1}{2}\,\bar{\mathrm {{H}}}_{F}\, g \big \Vert _{4}^2+ \big \Vert \textrm{A}_{F} -\frac{1}{2}\,\bar{\mathrm {{H}}}_{F}\, g \big \Vert _{2}^2\Big )\\&\le C \bigg [\bigg (\!\Big \Vert \textrm{A}_{F} -\frac{\bar{\mathrm {{H}}}_{F}}{2}\, g\Big \Vert _{2}\!\!+ \Vert \textrm{H}_{F}-\bar{\mathrm {{H}}}_{F} \Vert _{2}\!\bigg ) \Big \Vert \textrm{A}_{F} -\frac{\,\bar{\mathrm {{H}}}_{F}}{2} g\Big \Vert _{4}^2\!\!+\bigg (\!1\!+\Big \Vert \textrm{A}_{F} -\frac{\bar{\mathrm {{H}}}_{F}}{2}\, g\Big \Vert _{2}\bigg )\Big \Vert \textrm{A}_{F} -\frac{\,\bar{\mathrm {{H}}}_{F}}{2}\, g\Big \Vert _{2} \bigg ]. \end{aligned}$$

Since the estimates (4.1) and (4.6) imply that

$$\begin{aligned} \Vert \textrm{H}_{F}-\bar{\mathrm {{H}}}_{F} \Vert _{L^2(\partial F)} \! + \big \Vert \textrm{A}_{F} -\frac{1}{2}\,\bar{\mathrm {{H}}}_{F}\, g \big \Vert _{L^2(\partial F)}&\le C \big ( \Vert \textrm{H}_{F}-\bar{\mathrm {{H}}}_{F} \Vert _{L^2(\partial F)} \! + \big \Vert \mathrm {\text{\AA }}_{F} \big \Vert _{L^2(\partial F)} \big )\\&\le C {\bar{\varepsilon }}^{\frac{q}{2}} \end{aligned}$$

when ${\bar{\varepsilon }}>0$ is chosen small enough, we may absorb the term with $L^4$-norm on the right hand side with the one on left hand side and as a consequence, we get that

4.21 $$\begin{aligned} \Vert \textrm{A}_{F} -\frac{1}{2}\,\bar{\mathrm {{H}}}_{F}\, g \Vert _{L^4(\partial F)}^2 \le C {\bar{\varepsilon }}^{\frac{q}{2}}. \end{aligned}$$

Therefore using (4.3) and (4.21) the above implies that

$$\begin{aligned} \Vert \textrm{A}_{F}\Vert _{L^4(\partial F)}^4\le C\big (\Vert \textrm{A}_{F} -\frac{1}{2}\,\bar{\mathrm {{H}}}_{F}\, g \Vert _{L^4(\partial F)}^4+\bar{\mathrm {{H}}}_{F}^4 P(F)\big )\le C. \end{aligned}$$

Finally, (4.20) along with (4.21) gives inequality (4.15), and using (4.18) and (4.14), we obtain inequality (4.13).

*Step 2.2: We show that*

$$\begin{aligned} \max _{\partial F}|\textrm{H}_{F}-\bar{\mathrm {{H}}}_{F}|\le C{\bar{\varepsilon }}^{\frac{1}{8}}\,. \end{aligned}$$

The inequality (4.16) and the perimeter bound imply that we may use the interpolation inequalities from [41, Section 6] on $\partial F$. First, by [41, Proposition 6.2], we have

4.22 $$\begin{aligned} \max _{\partial F}|\textrm{H}_{F}-\bar{\mathrm {{H}}}_{F}|\le C\big (\Vert \nabla \textrm{H}_{F}\Vert _{L^4(\partial F)}+\Vert \textrm{H}_{F}-\bar{\mathrm {{H}}}_{F}\Vert _{L^4(\partial F)}\big )\,. \end{aligned}$$

To estimate the right hand side we apply [41, Proposition 6.5] and obtain

4.23 $$\begin{aligned} \Vert \nabla \textrm{H}_{F}\Vert _{L^4(\partial F)}&\le C \Vert \textrm{H}_{F}-\bar{\mathrm {{H}}}_{F}\Vert _{W^{2,2}(\partial F)}^{\frac{3}{4}}\, \Vert \textrm{H}_{F}-\bar{\mathrm {{H}}}_{F}\Vert _{L^2(\partial F)}^{\frac{1}{4}}, \end{aligned}$$

4.24 $$\begin{aligned} \Vert \textrm{H}_{F}-\bar{\mathrm {{H}}}_{F}\Vert _{L^4(\partial F)}&\le C \Vert \textrm{H}_{F}-\bar{\mathrm {{H}}}_{F}\Vert _{W^{1,2}(\partial F)}^{\frac{1}{2}}\, \Vert \textrm{H}_{F}-\bar{\mathrm {{H}}}_{F}\Vert _{L^2(\partial F)}^{\frac{1}{2}}. \end{aligned}$$

Note that the constants in (4.22), (4.23) and (4.24) are uniform, in particular, independent of $\varepsilon$.

In order to prove that

4.25 $$\begin{aligned} \Vert \textrm{H}_{F}-\bar{\mathrm {{H}}}_{F}\Vert _{W^{2,2}(\partial F)}\le C, \end{aligned}$$

we need a uniformly bound for $\Vert \nabla ^2 \textrm{H}_{F}\Vert _{L^{2}(\partial F)}$.

To this aim, we proceed in the spirit of [33, Lemma 2.5]. We multiply (2.8) by $\nabla \textrm{H}_{F}$ and integrate by parts to get that

4.26 $$\begin{aligned} \begin{aligned} \int _{\partial F} |\nabla ^2\textrm{H}_{F}|^2\, d {\mathcal {H}}^2&\le \int _{\partial F} (\Delta \textrm{H}_{F})^2\, d {\mathcal {H}}^2 + C\int _{\partial F} |\textrm{A}_{F}|^2\,|\nabla \textrm{H}_{F}|^2\,d {\mathcal {H}}^2\\&\le \int _{\partial F} (\Delta \textrm{H}_{F})^2\, d {\mathcal {H}}^2 +C\big ( 1 + \Vert \textrm{A}_{F} -\frac{1}{2}\,\bar{\mathrm {{H}}}_{F}\, g \Vert _{L^4(\partial F)}^2 \,\Vert \nabla \textrm{H}_{F}\Vert _{L^4(\partial F)}^2\big )\\&\le \int _{\partial F} (\Delta \textrm{H}_{F})^2\, d {\mathcal {H}}^2 +C\big ( 1 + {\bar{\varepsilon }}^{\frac{q}{2}} \,\Vert \nabla \textrm{H}_{F}\Vert _{L^4(\partial F)}^2\big ), \end{aligned} \end{aligned}$$

where we argue as in (4.20) and used the estimates (4.17) and (4.21). We use again the Euler-Lagrange equation (4.12) together with the inequalities (4.3), (4.13) and (2.9) and apply Young’s inequality and obtain that

$$ (\Delta \textrm{H}_{F})^2 \le C\big (1+|\textrm{A}_{F}|^6 \big ) \qquad \text {on } \, \partial F. $$

This and (4.4) yield that

4.27 $$\begin{aligned} \int _{\partial F} (\Delta \textrm{H}_{F})^2\, d {\mathcal {H}}^2\le C\Big (1+\int _{\partial F}|\textrm{A}_{F}|^6\,d {\mathcal {H}}^2\Big ). \end{aligned}$$

We apply [41, Proposition 6.1] with $p=1$ to the function $|\textrm{A}_{F}|^3$ and use Hölder’s inequality to obtain that

$$\begin{aligned} \left( \int _{\partial F}|\textrm{A}_{F}|^6\, d {\mathcal {H}}^2\right) ^{\frac{1}{2}}&\le C\Big (\int _{\partial F}|\textrm{A}_{F}|^2\, |\nabla \textrm{A}_{F}|\, d {\mathcal {H}}^2+\int _{\partial F}|\textrm{A}_{F}|^3\, d {\mathcal {H}}^2\Big )\\&\le C\Big (\Vert \textrm{A}_{F}\Vert _{L^4(\partial F)}^2\,\Vert \nabla \textrm{A}_{F}\Vert _{L^2(\partial F)}+\Vert \textrm{A}_{F}\Vert _{L^4(\partial F)}^3 \Big ) \end{aligned}$$

Therefore, inequality (4.15) along with (4.14) implies $\Vert \textrm{A}_{F}\Vert _{L^6(\partial F)}^6\le C$. Hence, we obtain

4.28 $$\begin{aligned} \int _{\partial F} (\Delta \textrm{H}_{F})^2\, d {\mathcal {H}}^2\le C. \end{aligned}$$

We apply [41, Proposition 6.1] to the function $\sqrt{|\nabla \textrm{H}_{F}|^4+\delta ^2}$, send $\delta \rightarrow 0$ and by the Young’s inequality with (4.17) we have

$$\begin{aligned} \Vert \nabla \textrm{H}_{F}\Vert _{L^4(\partial F)}^2 \le C \big (\Vert \nabla ^2 \textrm{H}_{F}\Vert _{L^2(\partial F)}^2 + \Vert \nabla \textrm{H}_{F}\Vert _{L^2(\partial F)}^2 \big ) \le C \big (1 +\Vert \nabla ^2 \textrm{H}_{F}\Vert _{L^2(\partial F)}^2\big ), \end{aligned}$$

which together with (4.26) and (4.28) yields

$$\begin{aligned} \Vert \nabla ^2 \textrm{H}_{F}\Vert _{L^2(\partial F)} \le C\big ( 1 + {\bar{\varepsilon }}^{\frac{q}{2}} \,\Vert \nabla ^2 \textrm{H}_{F}\Vert _{L^2(\partial F)}\big ). \end{aligned}$$

Therefore, when ${\bar{\varepsilon }}$ is small enough we obtain $\Vert \nabla ^2 \textrm{H}_{F}\Vert _{L^2(\partial F)} \le C$. In conclusion, the estimate (4.25) holds.

We use (4.1), (4.23), (4.24) and (4.25) to obtain

$$\begin{aligned} \Vert \nabla \textrm{H}_{F}\Vert _{L^4(\partial F)}\le C {\bar{\varepsilon }}^{\frac{1}{8}}\quad \quad \text {and}\quad \quad \Vert \textrm{H}_{F}-\bar{\mathrm {{H}}}_{F}\Vert _{L^4(\partial F)}\le C {\bar{\varepsilon }}^{\frac{1}{4}}\,. \end{aligned}$$

By (4.22), this implies that

4.29 $$\begin{aligned} \max _{\partial F}|\textrm{H}_{F}-\bar{\mathrm {{H}}}_{F}|\le C{\bar{\varepsilon }}^{\frac{1}{8}}\,. \end{aligned}$$

*Step 2.3: *$F$ *is the ball.* Let us write (4.29) in a slightly different way. We define that

$$ \textrm{H}_{F}^0=\frac{2P(F)}{3|F|}, $$

and notice that, by (4.3), (4.4) and $|F| = |B_1|,$ it holds that

$$ \bar{\mathrm {{H}}}_{F} - C {\bar{\varepsilon }}^{\frac{q}{2}} \le 2 \le \textrm{H}_{F}^0 \le \bar{\mathrm {{H}}}_{F} + C {\bar{\varepsilon }}^{\frac{q}{2}}. $$

Therefore we have, by (4.29), that

4.30 $$\begin{aligned} \big |\textrm{H}_{F}-\textrm{H}_{F}^0\big | \le C {\bar{\varepsilon }}^{\frac{q}{2}}. \end{aligned}$$

This in turn implies that

4.31 $$\begin{aligned} \delta _{\text {cmc}}(F):=\Big \Vert \frac{\textrm{H}_{F}}{\textrm{H}_{F}^0}-1\Big \Vert _{C^0(\partial F)}\le C {\bar{\varepsilon }}^{\frac{q}{2}}. \end{aligned}$$

Notice that the quantity $\delta _{\text {cmc}}(\cdot )$ is scaling invariant, while $\textrm{H}_{F}^0$ is not. Therefore, scaling $F$ such that $\textrm{H}_{\alpha F}^0=2$ and possibly choosing a smaller ${\bar{\varepsilon }}>0$, the assumptions of [30, Theorem 1.8] are satisfied by $\widetilde{F}=\alpha F$, where $\alpha =P(F)/P(B_1)$. Accordingly, by the proof of [30, Theorem 1.8] and the arguments in [48], there exists $\widetilde{w}\in C^{1,1}(\mathbb {S}^2)$ such that, up to translation, the barycenter of ${\widetilde{F}}$ is the origin and $\partial \widetilde{F}=\{(1+\widetilde{w}(x))x:\,x\in \mathbb {S}^2\}$ with $\Vert \widetilde{w}\Vert _{C^{1}(\mathbb {S}^2)}\le C\,\delta _{\text {cmc}}(F)$. Thus,

$$\begin{aligned} \partial F=\{(1+w(x))x\,:\,x\in \mathbb {S}^2\}\quad \text {with}\quad w(x)=\frac{1}{\alpha }\Big [\Big (1-\alpha \Big )+\widetilde{w}\Big ] \end{aligned}$$

and

$$\begin{aligned} \Vert w\Vert _{C^{1}(\mathbb {S}^2)}\le |1-\alpha |+\Vert \widetilde{w}\Vert _{C^{1}(\mathbb {S}^2)}\le C{\bar{\varepsilon }}^{\,\widetilde{q}}\, \end{aligned}$$

for some $\widetilde{q}\in (0,1)$, by using (4.4) and its consequence $\alpha ^{-1}\le 1$. We may now use the quantitative Alexandrov theorem from [48] for nearly spherical sets, by possibly decreasing ${\bar{\varepsilon }}>0$, which implies

$$\begin{aligned} P(F) - P(B_1) \le C \Vert w\Vert _{W^{1,2}(\mathbb {S}^2)}^2 \le C\Vert \textrm{H}_{F}-\bar{\mathrm {{H}}}_{F} \Vert _{L^2(\partial F)}^2. \end{aligned}$$

Thus, we have obtained $P(F) - P(B_1) \le C_1 \Vert \textrm{H}_{F}-\bar{\mathrm {{H}}}_{F} \Vert _{L^2(\partial F)}^2$ for a uniform constant $C_1>0$. Therefore, when ${\bar{\varepsilon }}<1/C_1$, it holds that

$$\begin{aligned} J_\varepsilon (F) = \Vert \textrm{H}_{F}-\bar{\mathrm {{H}}}_{F} \Vert _{L^2(\partial F)}^2 - \varepsilon P(F) \ge - \varepsilon P(B_1) = J_\varepsilon (B_1) \end{aligned}$$

and the equality is true only when $F$ is the ball. By the minimality of $F$ we have equality in the above and thus $F$ is the ball. $\square$

We are now ready to prove Theorem 1.1, which follows easily from Proposition 4.1, as we will see below.

### Proof of Theorem 1.1

Let ${\bar{\varepsilon }}>0$ be from Proposition 4.1 and fix $\varepsilon \in (0,{\bar{\varepsilon }})$. For every $C^2$-regular $E\subset \mathbb {R}^3$ satisfying the conditions (1.1), by Proposition 4.1, we know that $J_\varepsilon (E)\ge J_\varepsilon (B_1)$, which implies

$$\begin{aligned} P(E)-P(B_1)\le \varepsilon ^{-1\,}\Vert \textrm{H}_{E}-\bar{\mathrm {{H}}}_{E} \Vert _{L^2(\partial E)}^2\,. \end{aligned}$$

The rest of the statement of Theorem 1.1 follows from Corollary 3.3. $\square$

### Remark 4.2

By a simple scaling argument we have the statement of Theorem 1.1 for sets with generic volume $|E| = v$. Indeed, fix $v>0$ and denote the radius $\rho$ with $|B_\rho | = v$. By Theorem 1.1 for every $\delta _0>0$ there exists $C(\delta _0)>0$ such that for every $C^2$-regular set $E\subset \mathbb {R}^3$ with $|E|=|B_\rho |$ and $P(E) \le (4\pi \root 3 \of {2} - \delta _0)\rho ^2$ it holds that

4.32 $$\begin{aligned} \big |P(E)- 4 \pi \rho ^2\big |\le C \rho ^2 \, \big \Vert \textrm{H}_{E}-\bar{\mathrm {{H}}}_{E}\big \Vert _{L^2(\partial E)}^2. \end{aligned}$$

## Application to Volume Preserving Curvature Flow

We begin this section by proving the following consequence of Theorem 1.1:

### Proposition 5.1

Assume that there are balls $B_{r}(x_1), \dots , B_{r}(x_N)$ which are quantitatively disjoint in the sense that $|x_i - x_j|\ge 2r +\delta _1$ with $i \ne j$ for some $\delta _1 >0$ such that their union $F = \bigcup _{i=1}^N B_{r}(x_i)$ has the measure $|F| = |B_1|$. Then there is $\varepsilon _0= \varepsilon _0(\delta _1, M)>0$ such that for all $C^2$-regular sets $E \subset \mathbb {R}^3$ with $|E|= |B_1|$, $P(E)\le M$ and $|E \Delta F|\le \varepsilon _0$ it holds that

$$ P(E) - 4 \pi N^{\frac{1}{3}} \le C \Vert \textrm{H}_{E}-\bar{\mathrm {{H}}}_{E}\Vert _{L^2(\partial E )}^2, $$

for a constant *C* that depends on *M* and $\delta _1>0$.

### Proof

We note that we may assume that $\Vert \textrm{H}_{E}-\bar{\mathrm {{H}}}_{E}\Vert _{L^2(\partial E )} \le \varepsilon _1$, for small $\varepsilon _1>0$, since otherwise the claim is trivially true for $C\ge M/\varepsilon _1$. Then by Theorem 3.1 and (3.1) there exists a union of disjoint equisize balls, denote it by ${\tilde{F}}$, such that $|{\tilde{F}}| = |B_1|$ and

$$ \sup _{x \in E \Delta {\tilde{F}}} |d_{{\tilde{F}}}| + \big | P(E) - P({\tilde{F}}) \big |\le C \varepsilon _1^q. $$

By the assumption $|E \Delta F|\le \varepsilon _0$, where $F = \bigcup _{i=1}^N B_r(x_i)$ with $|x_i-x_j|\ge 2 r + \delta _1$ for $i \ne j$, the sets *F* and ${\tilde{F}}$ must both have *N* many balls with the same radius *r* and we have

5.1 $$\begin{aligned} \sup _{x \in E \Delta F} |d_{F}| + \big | P(E) - 4 \pi N^{\frac{1}{3}} \big | \le C \varepsilon _0, \end{aligned}$$

when $\varepsilon _1$ is chosen small. Note that $r= N^{-\frac{1}{3}}$ and *N* is bounded by the perimeter bound on *E*. From the above we deduce that there are disjoint sets $E_1, \dots , E_N$ such that $E = \bigcup _{i=1}^N E_i$ and $B_{r-C\varepsilon _0}(x_i) \subset E_i \subset B_{r+C\varepsilon _0}(x_i)$. Let us choose for every *i* radius $r_i$ such that $|E_i| = |B_{r_i}|$. Then $r-C\varepsilon _0 \le r_i \le r+C\varepsilon _0$ and by the isoperimetric inequality

$$ P(E_i) \ge 4 \pi r_i^2\ge 4 \pi (r- C \varepsilon _0)^2 \ge 4 \pi N^{-\frac{2}{3}} - C\varepsilon _0. $$

On the other hand by (5.1) it holds that

$$ P(E) = \sum _{i =1}^N P(E_i) \le 4 \pi N^{\frac{1}{3}} + C \varepsilon _1^q. $$

Therefore it holds $|P(E_i) - 4 \pi r_i^2| \le C\varepsilon _0$ for all $i = 1, \dots , N$ and (4.32) implies

$$ P(E_i) - 4 \pi r_i^2 \le C \Vert \textrm{H}_{E_i}-\bar{\mathrm {{H}}}_{E_i}\Vert _{L^2(\partial E_i)}^2 \le C \Vert \textrm{H}_{E_i}-\bar{\mathrm {{H}}}_{E}\Vert _{L^2(\partial E_i)}^2. $$

Summing over *i* yields that

$$ P(E) - 4 \pi \sum _{i=1}^N r_i^2 \le C \sum _{i=1}^N \Vert \textrm{H}_{E_i}-\bar{\mathrm {{H}}}_{E}\Vert _{L^2(\partial E)}^2 = C \Vert \textrm{H}_{E}-\bar{\mathrm {{H}}}_{E}\Vert _{L^2(\partial E)}^2. $$

Recall that the radii $r_i$ are such that $\sum _{i=1}^N |B_{r_i}| = |B_1|$, i.e., $\sum _{i=1}^N r_i^3 = 1$. Therefore it holds that

$$ 4 \pi \sum _{i=1}^N r_i^2 \le 4 \pi \left( \sum _{i=1}^N r_i^3 \right) ^{\frac{2}{3}} \left( \sum _{i=1}^N 1 \right) ^{\frac{1}{3}} =4 \pi N^{\frac{1}{3}}, $$

which implies the claim. $\square$

In this section we will show how the sharp quantitative Alexandrov theorem, i.e., Theorem 1.1, implies the convergence of the flat flow for the volume preserving mean curvature flow (1.4). We begin by recalling the definition of the flat flow. This was first given in [50], following the associated scheme proposed in [3, 40], but here we use the variant given in [23] since it simplifies the forthcoming analysis. We refer to [23, 48, 50] for a more detailed introduction.

We fix the time step $h>0$, and given a bounded set of finite perimeter *E* with $|E| = v\in (0,+\infty )$, we consider the minimization problem

5.2 $$\begin{aligned} \min \Big \{ P(F) + \frac{1}{h}\int _F d_E \, dx : \, |F| = v \Big \}, \end{aligned}$$

and note that the minimizer exists but might not be unique. Here $d_E$ denotes the signed distance function as defined in (2.1). We define the dissipation of a set *F* with respect to a set *E* as

5.3 $$\begin{aligned} \mathcal {D}(F,E) := \int _{F \Delta E} dist (x,\partial E)\, dx, \end{aligned}$$

and observe that we may write the minimization problem (5.2) as

5.4 $$\begin{aligned} \min \Big \{ P(F) + \frac{1}{h}\mathcal {D}(F,E) : \, |F| = v \Big \}. \end{aligned}$$

We note that by a simple scaling argument we may reduce to the case $v = |B_1|$ by changing the value of *h*.

Let $E(0) \subset \mathbb {R}^3$ then be a bounded set of finite perimeter with $|E(0)|= |B_1|$ and which coincides with its Lebesgue representative. We construct the discrete-in-time evolution $\{E_k^{(h)}\}_{k\in \mathbb {N}}$ by recursion in such a way that $E(0)=E$ and assuming that $E_k^{(h)}$ is defined we set $E_{k+1}^{(h)}$ to be a minimizer of (5.2) with $E= E_k^{(h)}$. Notice that by standard regularity theory $\partial E_k^{(h)}$ is $C^{2,\alpha }$-regular (for all $\alpha \in (0,1)$) and therefore in (5.4) we use this exact representative in order to compute $dist (\cdot ,\partial E_k^{(h)})$. We define the *approximate volume preserving flat flow* $\{E^{(h)}(t)\}_{t \ge 0}$ by setting that

$$ E^{(h)}(t) = E_k^{(h)} \qquad \text {for }\, t \in [kh, (k+1) h). $$

Let us then recall some basic a priori estimates for the approximative flat flow $\{E^{(h)}(t)\}_{t \ge 0}$. Using the minimality of $E_{k+1}^{(h)}$ and comparing its energy (5.2) against the previous set $E_k^{(h)}$, we obtain the following important estimate for all $k =0,1, \dots :$

5.5 $$\begin{aligned} P(E_{k+1}^{(h)}) + \frac{1}{h}\mathcal {D}(E_{k+1}^{(h)},E_k^{(h)}) \le P(E_{k}^{(h)}) . \end{aligned}$$

In view of the feat that $\partial E_{k+1}^{(h)}$ is $C^{2,\alpha }$-regular (for all $\alpha \in (0,1)$), This satisfies the Euler-Lagrange equation

5.6 $$\begin{aligned} \frac{d_{E_k^{(h)}}}{h} = - \textrm{H}_{E_{k+1}^{(h)}} + \lambda _{k+1}^{(h)} \qquad \text {on }\, \partial E_{k+1}^{(h)}, \end{aligned}$$

in the classical sense, where $\lambda _{k+1}^{(h)}$ is the Lagrange multiplier due to the volume penalization. It follows from the argument in the proof of [50, Lemma 3.6] or [23, Lemma 4.2], that

5.7 $$\begin{aligned} \int _{ \partial E_{k+1}^{(h)}} d_{E_{k}^{(h)}}^2 \, d {\mathcal {H}}^2 \le C \mathcal {D}(E_{k+1}^{(h)},E_k^{(h)}) \end{aligned}$$

for some universal constant $C>0$. By summing (5.5) from $k =0$ to $k \rightarrow \infty$, and using (5.6) and (5.7), we obtain the dissipation inequality

5.8 $$\begin{aligned} \int _{h}^\infty \int _{ \partial E^{(h)}(t)} (\textrm{H}_{E^{(h)}(t)} - \bar{\mathrm {{H}}}_{E^{(h)}(t)})^2 \, d {\mathcal {H}}^2 dt \le C P(E(0)) . \end{aligned}$$

Note also that (5.5) implies that $t\mapsto P(E^{(h)}(t))$ is non-increasing. We now recall the definition of *flat flow*.

### Definition 5.2

A flat flow solution of (1.4) is any family of sets $\{E(t)\}_{t \ge 0}$ which is a cluster point of $\{E^{(h)}(t)\}_{t \ge 0}$, i.e.,

$$ E^{(h_n)}(t) \rightarrow E(t) \quad \text {as } \, h_n \rightarrow 0 \quad \text {in } \, L^1 \quad \text {for almost every }\, t >0 . $$

By [23, Theorem 1] there exists a flat flow starting from *E*(0).

We are interested in the long time behavior of the flow. To this end, we recall the following elementary algebraic lemma from [24],

### Lemma 5.3

(Discrete Grönwall’s Inequality) Let $K\in \mathbb {N}$ and $\{a_k\}_{k\in \{1,\ldots , K\}}$ be a sequence of non-negative numbers. Assume that there exists $C>1$ such that

$$ \sum _{k=i}^{K}a_k\le C a_i $$

for every $i\in \{1,\ldots , K\}$. Then,

$$ \sum _{k=i+1}^{K}a_k\le \Bigl (1-\frac{1}{C}\Bigr )^{i}S $$

for every $i\in \{1,\ldots , K-1\}$, where $S:=\sum _{k=1}^{K}a_k$.

The next lemma is a particular case of the well-known fact that $C^2$-sets are $\Lambda$-minimizers of the perimeter (see e.g. [1, Lemma 4.1]).

### Lemma 5.4

Let $N \in \mathbb {N}$ and $B_r(x_1), \dots , B_r(x_N)$ be disjoint balls in $\mathbb {R}^n$, with $|x_i-x_j|\ge 2r+ \delta _1$ for $i\ne j$, and denote $F = \bigcup _{i=1}^N B_r(x_i)$. Then there exists a constant $\Lambda >0$, which depends on *r*, *n*, *N* and $\delta _1$, such that for every set of finite perimeter $E \subset \mathbb {R}^n$ it holds that

$$ P(F) \le P(E) + \Lambda |E\Delta F|. $$

### Proof of Theorem 1.2

Let $\{E^{h_n}(t)\}_{t\ge 0}$ an approximate flat flow converging to $\{E(t)\}_{t\ge 0}$ and let *N*, $r>0$, and $x_i(t)$, $i=1, \dots , N$, be as in the statement. By rescaling we may assume that $|E(0)| = |B_1|$. Set that

$$ f_n(t) = P(E^{(h_n)}(t)). $$

By (5.5) the $f_n$’s are monotone non-increasing functions which are bounded by *P*(*E*(0)). Therefore, by Helly’s Selection Theorem, up to passing to a further sub-sequence (not relabeled), we may assume that the functions $f_n$’s converge pointwise to some non-increasing function $f_\infty :[0,+\infty )\rightarrow \mathbb {R}$. Note that it follows from (1.6) and Lemma 5.4 that

$$ \lim _{t \rightarrow \infty }f_\infty (t) \ge 4 \pi N^{\frac{1}{3}}. $$

We need to distinguish two cases.

**Case 1.** We first assume that

5.9 $$\begin{aligned} f_\infty (t)> 4\pi N^{\frac{1}{3}} \quad \text {for all }t\in [0, +\infty ). \end{aligned}$$

We observe that by using the argument in [25], i.e., using the estimates (4.23) and (4.25) in the proof of [25, Theorem 1.1], we have that for every $\varepsilon >0$ there exists $T_\varepsilon >1$ with the following property: for every $T>T_\varepsilon$ there exists $h_0=h_0(\varepsilon , T)>0$ such that for $0<h_n\le h_0$ and $t\in (T_\varepsilon , T)$ there exist points $x^{(h_n)}_1(t)$, ..., $x^{(h_n)}_N(t)$ satisfying

- $$|x^{(h_n)}_i(t)-x^{(h_n)}_j(t)|\ge 2r$$ and $$x^{(h_n)}_i(t)\rightarrow x_i(t)$$ as $$h_n\rightarrow 0$$,
- $\displaystyle \sup _{ E^{(h_n)}(t)\Delta F^{(h_n)}(t)}dist (\cdot , \partial F^{(h_n)}(t))\le \varepsilon$, where we have set $\displaystyle F^{(h_n)}(t):=\bigcup _{i=1}^NB_r\big (x^{(h_n)}_i(t)\big )$.

Recall now that $|E^{(h_n)}(\cdot )\Delta E(\cdot )|\rightarrow 0$ uniformly in $(T_\varepsilon , T)$, as $E^{(h_n)}(\cdot )$ and $E(\cdot )$ are Hölder continuous [23, 25, 50], and recall that (1.6) holds. Hence by choosing $T_\varepsilon$ larger and $h_0$ smaller, we may also ensure (by triangular inequality) that $F^{(h_n)}(t)$ is uniformly $(C\varepsilon )$-close to *F*(*t*) in $(T_\varepsilon , T)$ with respect to the $L^1$-distance, and thus with respect to the Hausdorff distance up to change the constant *C*, which is always independent both of *n* and *T*. Hence, by choosing $\varepsilon$ sufficiently small and recalling (1.7), we may assume that, in fact,

$$ |x^{(h_n)}_i(t)-x^{(h_n)}_j(t)|\ge 2r+\frac{\delta _1}{2}\quad \text { for }0<h_n\le h_0\text { and }t\in (T_\varepsilon , T). $$

We may thus use Proposition 5.1 to deduce that

5.10 $$\begin{aligned} P(E^{(h_n)}(t)) - 4 \pi N^{\frac{1}{3}} \le C \Vert \textrm{H}_{E^{(h_n)}(t)}-\bar{\mathrm {{H}}}_{E^{(h_n)}(t)}\Vert _{L^2(\partial E^{(h_n)}(t) )}^2 \end{aligned}$$

for a constant that depends on *P*(*E*(0)) and $\delta _1$.

We proceed by proving that the *E*(*t*) converges in $L^1$-sense. The argument is now similar to that of [24, Theorem 1.2] but some modifications are needed. In what follows *C* will denote a positive constant independent both of *n* and *T*, which may change from line to line and also within the same display.

Let $T>T_\varepsilon$ be as above. Then, by (5.9) and the definition of $f_\infty$, it follows that

$$ P(E^{(h_n)}(t))> 4\pi N^{\frac{1}{3}}\quad \text {for all }t\in [0, T] $$

and for $0<h_n\le h_0$ (by taking $h_0>0$ smaller if needed). In turn (for the same *n*’s), by iterated use of (5.5) and then by (5.10) we deduce for all $t\in (T_\varepsilon +h_n, T-h_n)$

$$\begin{aligned} \frac{1}{h_n}\sum _{k= \lfloor \frac{t}{h_n}\rfloor }^{\lfloor \frac{T}{h_n}\rfloor -1} \mathcal {D}(E_{k+1}^{(h_n)},E_{k}^{(h_n)})&\le P(E^{(h_n)}(t)) -P(E^{(h_n)}(T)) < P(E^{(h_n)}(t)) - 4\pi N^{\frac{1}{3}} \\&\le {\widetilde{C}} \Vert \textrm{H}_{E^{(h_n)}(t)}-\bar{\mathrm {{H}}}_{E^{(h_n)}(t)}\Vert _{L^2}^2, \end{aligned}$$

where $\lfloor a\rfloor$ denotes the integer part of $a >0$.

We proceed by using the Euler-Lagrange equation (5.6) and the estimate (5.7) to get

5.11 $$\begin{aligned} \begin{aligned} \Vert \textrm{H}_{E^{(h_n)}(t)}-\bar{\mathrm {{H}}}_{E^{(h_n)}(t)}\big \Vert _{L^2}^2&\le \Vert \textrm{H}_{E^{(h_n)}(t)}- \lambda _{\lfloor \frac{t}{h_n}\rfloor }^{(h_n)} \big \Vert _{L^2}^2 \\&=\frac{1}{h_n^2}\Vert d_{E^{(h_n)}(t-h_n)} \big \Vert _{L^2(\partial E^{(h_n)}(t))}^2 \le \frac{C}{h_n^2}\mathcal {D}(E_{\lfloor \frac{t}{h_n}\rfloor }^{(h_n)},E_{\lfloor \frac{t}{h_n}\rfloor -1}^{(h_n)}). \end{aligned} \end{aligned}$$

The two previous displays yield

$$ \frac{1}{h_n} \sum _{k=\lfloor \frac{t}{h_n}\rfloor -1 }^{\lfloor \frac{T}{h_n}\rfloor -1} \mathcal {D}(E_{k+1}^{(h_h)},E_{k}^{(h_n)}) \le \frac{C+h_n}{h_n^2} \mathcal {D}(E_{\lfloor \frac{t}{h_n}\rfloor }^{(h_n)},E_{\lfloor \frac{t}{h_n}\rfloor -1}^{(h_n)})\le \frac{C'}{h_n^2} \mathcal {D}(E_{\lfloor \frac{t}{h_n}\rfloor }^{(h_n)},E_{\lfloor \frac{t}{h_n}\rfloor -1}^{(h_n)}), $$

with $C'>0$ independent of *T* and *n*. Denoting $a_k = \frac{1}{h_n} \mathcal {D}(E_{k+1}^{(h_h)},E_{k}^{(h_n)})$ and recalling that

$$ \frac{1}{h_n} \sum _{k=\lfloor \frac{T_\varepsilon }{h_n}] }^{\lfloor \frac{T}{h_n}\rfloor -1} \mathcal {D}(E_{k+1}^{(h_h)},E_{k}^{(h_n)})\le P(E^{(h_n)}(T_\varepsilon ))\le P(E(0)), $$

we may use Lemma 5.3 to deduce that

5.12 $$\begin{aligned} \frac{1}{h_n} \sum _{k=\lfloor \frac{t}{h_n}\rfloor }^{\lfloor \frac{T}{h_n}\rfloor -1} \mathcal {D}(E_{k+1}^{(h_h)},E_{k}^{(h_n)}) \le P(E(0)) \left( 1 - \frac{h_n}{C'}\right) ^{\lfloor \frac{t}{h_n}\rfloor -\lfloor \frac{T_\varepsilon }{h_n}\rfloor }\le C e^{-t/C'} \end{aligned}$$

for all $t\in (T_\varepsilon +h_n, T-h_n)$ and $0<h_n\le h_0$.

By [50, Proposition 3.4] it holds that

$$ |E^{(h_n)}_{k+1}\Delta E^{(h_n)}_{k}| \le C l P(E^{(h_n)}_{k+1}) + \frac{C}{l} \mathcal {D}(E_{k+1}^{(h_n)},E_{k}^{(h_n)}) $$

for all $l \le \frac{1}{C} \sqrt{h_n}$. Therefore, by the inequality above, by $P(E^{(h_n)}_{k+1}) \le P(E(0))$ and by (5.12) for every *t*, *s*, with $T_\varepsilon + h_n \le t < s \le t+1 \le T-h_n$, we have that

5.13 $$\begin{aligned} \begin{aligned} |E^{(h_n)}(t)\Delta E^{(h_n)}(s)|&\le \sum _{k=\lfloor \frac{t}{h_n}\rfloor }^{\lfloor \frac{s}{h_n}\rfloor -1} |E^{(h_n)}_{k+1}\Delta E^{(h_n)}_{k}|\\&\le C P(E(0)) l \frac{s-t}{h_n} +\frac{C}{l}\sum _{k=\lfloor \frac{t}{h_n}\rfloor }^{\lfloor \frac{s}{h_n}\rfloor -1}\mathcal {D}(E^{(h_n)}_{k+1},E^{(h_n)}_{k})\\&\le \frac{C l}{h_n} +\frac{C \, h_n}{l} e^{-t/C'}, \end{aligned} \end{aligned}$$

for all $l \le \frac{1}{C} \sqrt{h_n}$ and $0<h_n\le h_0$. In particular, choosing $l= h_n e^{-t/2C'}$, we have

$$ |E^{(h_n)}(t)\Delta E^{(h_n)}(s)| \le Ce^{-t/2C'}. $$

Passing to the limit as $h_n\rightarrow 0$, we get that

5.14 $$\begin{aligned} |E(t)\Delta E(s)| \le Ce^{-t/2C'} \qquad \text {for all} \, \, T_\varepsilon \le t < s \le t+1 \le T. \end{aligned}$$

Since $T>T_\varepsilon$ is arbitrary, we conclude that *E*(*t*) converges exponentially fast to a set of finite perimeter *F* in $L^1$, which by our assumptions is necessarily of the form

5.15 $$\begin{aligned} F = \bigcup _{i =1}^N B_r(x_i), \quad \text {with }|x_i -x_j| \ge 2r + \delta _1\text { for }i \ne j. \end{aligned}$$

Let us also observe that for, $t\in (T_\varepsilon +h_n, T-h_n)$ and $0<h_n\le h_0$, we have

5.16 $$\begin{aligned} \begin{aligned} \int _{t+h_n}^{T-h_n} \Vert \textrm{H}_{E^{(h_n)}(s)}&-\bar{\mathrm {{H}}}_{E^{(h_n)}(s)}\big \Vert _{L^2(\partial E^{(h_n)}(s))}^2\, ds \le \sum _{k= \lfloor \frac{t}{h_n}\rfloor }^{\lfloor \frac{T}{h_n}\rfloor -1} h_n \Vert \textrm{H}_{E_{k+1}^{(h_n)}}-\bar{\mathrm {{H}}}_{E_{k+1}^{(h_n)}}\big \Vert _{L^2(\partial E_{k+1}^{(h_n)})}^2 \\&\le \frac{C}{h_n} \sum _{k=\lfloor \frac{t}{h_n}\rfloor }^{\lfloor \frac{T}{h_n}\rfloor -1} \mathcal {D}(E_{k+1}^{(h_h)},E_{k}^{(h_n)}) \le C e^{-t/C'} \end{aligned} \end{aligned}$$

where in the second inequality we argued as in (5.11), while in the last we used (5.12).

From (5.16) and from the mean value theorem, we deduce that for any $t \in [T_\varepsilon +1, T-1]$ and $0<h_n\le h_0$ there exists $t_n \in [t - e^{-t/2C'}, t]$ such that

$$ \Vert \textrm{H}_{E^{(h_n)}(t_n)} -\bar{\mathrm {{H}}}_{E^{(h_n)}(t_n)}\big \Vert _{L^2(\partial E^{(h_n)}(t_n))}^2 \le C e^{-t/2C'} . $$

By Theorem 3.1 and (3.1) we deduce that $E^{(h_n)}(t_n)$ is close to a set $F^{(h_n)}(t_n):=\bigcup _{i=1}^N B_r(x_i^{(h_n)}(t_n) )$ for suitable $x_i^{(h_n)}(t_n) \in \mathbb {R}^3$, not only in the $L^1$-sense, but also in the same that

5.17 $$\begin{aligned} \sup _{x\in E^{(h_n)}(t_n) \Delta F^{(h_n)}(t_n) }dist (\cdot , \partial F^{(h_n)}(t_n)) + P(E^{(h_n)}(t_n)) - 4 \pi N^{\frac{1}{3}} \le {C e^{- qt/2C'}.} \end{aligned}$$

Since $t_n\le t$ we have $P(E^{(h_n)}(t_n)) \ge P(E^{(h_n)}(t))$ and thus (5.17) holds a fortiori with $P(E^{(h_n)}(t_n))$ replaced by $P(E^{(h_n)}(t))$. Therefore sending $h_n\rightarrow 0$, we deduce by the lower semicontinuity of the perimeter that

5.18 $$\begin{aligned} P(E(t)) - 4 \pi N^{\frac{1}{3}} \le {C e^{- qt/2C'}.} \end{aligned}$$

On the other hand, by Lemma 5.4, we have

5.19 $$\begin{aligned} 4 \pi rN^{\frac{1}{3}}= P(F)\le P(E(t))+ \Lambda |E(t)\Delta F|, \end{aligned}$$

with *F* given by (5.15). Recalling that $|E(t)\Delta F|\rightarrow 0$ exponentially fast, (5.18) and (5.19) yield the exponential convergence of *P*(*E*(*t*)) to $N4 \pi r^2$ as $t\rightarrow +\infty$.

We now turn to the Hausdorff convergence. Notice that inequality (5.17) is true for *t*, possibly with a worse exponential rate, by applying [25, Lemma 4.3], and observe that passing to the limit in (5.17) (up to a *t*-dependent further sub-sequence, if needed) yields

5.20 $$\begin{aligned} \sup _{ E(t) \Delta F(t)} dist (\cdot , \partial F(t)) \le {C e^{- qt/C'} ,} \end{aligned}$$

where $F(t):=\bigcup _{i=1}^N B_r(x_i(t))$ for suitable $x_i(t) \in \mathbb {R}^3$. Recalling that $|E(t)\Delta F|\rightarrow 0$ exponentially fast as $t\rightarrow +\infty$, we deduce that also *F*(*t*) converges to *F* exponentially fast (with respect to the $L^1$-distance and hence the Hausdorff distance) and thus (5.20) holds also with *F*(*t*) replaced by *F*, possibly with a worse exponential rate.

**Case 2.** Assume that here exists ${\bar{t}}> 0$ such that $f_\infty (t) = 4 \pi N^{\frac{1}{3}}$ for every $t\ge {\bar{t}}$. In this case we will show that for large times *E*(*t*) *coincides* with *F*. The argument is identical to that of [24, Theorem 1.2-Case 2], but we reproduce it have for the reader’s convenience.

By monotonicity of the functions $f_n$’s, we have that for every $T>{\bar{t}}$, $\{f_n\}$ converges uniformly to $f_\infty \equiv 4 \pi N^{\frac{1}{3}}$ in $[{\bar{t}}, T]$. In particular, using that

$$ \frac{1}{h_n} \mathcal {D}(E^{(h_n)}_{k+1}, E^{(h_n)}_{k}) \le f_n(k h_n) - f_n((k+1) h_n), $$

we deduce, for every $t\in [{\bar{t}}, T-h_n]$, that

$$ \sum _{k=\lfloor \frac{t}{h_n}\rfloor }^{\lfloor \frac{T}{h_n}\rfloor -1} h_n^{-1}\mathcal {D}(E^{(h_n)}_{k+1},E^{(h_n)}_{k}) \le f_n\Big (\lfloor \frac{t}{h_n}\rfloor h_n\Big ) - f_n\Big (\lfloor \frac{T}{h_n}\rfloor h_n\Big )=:b_n \rightarrow 0 \qquad as h_n\rightarrow 0. $$

Arguing as in (5.13), for every ${\bar{t}} \le t < s \le T-h_n$, we get

$$\begin{aligned} |E^{(h_n)}(t)\Delta E^{(h_n)}(s)| \le C l \frac{s-t}{h_n} P(E(0))+\frac{C}{l}\sum _{k=[\frac{t}{h_n}] }^{\lfloor \frac{s}{h_n}\rfloor -1}\mathcal {D}(E^{(h_n)}_{k+1},E^{(h_n)}_{k}), \end{aligned}$$

for all $l \le \frac{1}{C} \sqrt{h_n}$. Choosing $\ell = \sqrt{b_n} h_n$, we conclude that;

$$\begin{aligned} |E^{(h_n)}(t)\Delta E^{(h_n)}(s)| \le C \sqrt{b_n} (s-t) P(E(0))+C \sqrt{b_n} \rightarrow 0, \end{aligned}$$

that is, $E(t) = E(s)$, for every ${\bar{t}}<t<s<T$. By the arbitrariness of $T>{\bar{t}}$ and recalling that by assumption *E*(*t*) converges to a disjoint union of balls up to translations, we necessarily have $E(t)=F$ for all $t\ge {\bar{t}}$, with *F* as in (5.15). $\square$

## The Asymptotics of the 3D Mullins–Sekerka Flow

Let us first construct a flat flow solution for the Mullins–Sekerka flow (1.8) in the three-dimensional flat torus $\mathbb {T}_R^3 = \mathbb {R}^3/(R\mathbb {Z}^3)$ with $R \ge 1$. The construction in the case of a bounded domain is due to Luckhaus and Sturzenhecker [40], while the two dimensional flat torus $\mathbb {T}^2$ is considered in [24]. The construction in $\mathbb {T}_R^3$ is essentially the same as in the two-dimensional case but we repeat it for the reader’s convenience. First, the perimeter of *E* in the flat torus $\mathbb {T}_R^3$ is defined as

$$ P_{\mathbb {T}_R^3}(E) := \sup \Big \{ \int _E \textrm{div}X \, dx : X \in C^1(\mathbb {T}_R^3,\mathbb {R}^3) , \, \Vert X\Vert _{L^\infty } \le 1 \Big \}. $$

Here $X \in C^1(\mathbb {T}_R^3,\mathbb {R}^2)$ means that the $\mathbb {Z}_R^3$-periodic extension of *X* to $\mathbb {R}^3$ is continuously differentiable. For a given set of finite perimeter $E \subset \mathbb {T}_R^3$, with $|E|=v$, we consider the minimization problem

6.1 $$\begin{aligned} \min \Big \{ P_{\mathbb {T}_R^3}(F) + \frac{h}{2} \int _{\mathbb {T}_R^3} |D \, U_{F,E}|^2 \, dx :\quad \text {with }\, |F| = |E| = v \Big \}, \end{aligned}$$

where the function $U_{F,E}\in H^1(\mathbb {T}_R^3)$ is the solution of

6.2 $$\begin{aligned} -\Delta U_{F,E} = \frac{1}{h} \left( \chi _F - \chi _E\right) \end{aligned}$$

with zero average. We note first that by a simple scaling argument we may reduce to the case $v = |B_1|$ by changing the values of *R* and *h*. As proven in [40, 57] there exists a minimizer for (6.1), but it might not be unique. Concerning the regularity of the minimizers, we may argue as in [24] to deduce that the minimizing set *F* is $C^{3,\alpha }$-regular and satisfies the associated Euler-Lagrange equation

$$ U_{F,E} = -\textrm{H}_{F} + \lambda \qquad \text {on }\, \partial F $$

in the classical sense, where $\lambda$ is the Lagrange multiplier due to the volume constraint.

We denote that

6.3 $$\begin{aligned} \mathfrak {D}(F,E) := \int _{\mathbb {T}_R^3} |D \, U_{F,E}|^2 \, dx , \end{aligned}$$

where $U_{F,E}$ is defined in (6.2). We define the $H^{-1}$-norm of a function *f* on the torus $\mathbb {T}_R^3$ by duality as

$$ \Vert f\Vert _{H^{-1}(\mathbb {T}_R^3)} := \sup \Big \{ \int _{\mathbb {T}_R^3} \varphi \, f \, dx : \Vert D \varphi \Vert _{L^2(\mathbb {T}_R^3)} \le 1\Big \} . $$

Then, by (6.2) and integration by parts, we have that

6.4 $$\begin{aligned} \Vert \chi _F - \chi _E\Vert _{H^{-1}(\mathbb {T}_R^3)}^2 \le h^2 \, \Vert D \, U_{F,E} \Vert _{L^2(\mathbb {T}_R^3)}^2 = h^2 \, \mathfrak {D}(F,E). \end{aligned}$$

We fix the time step $h >0$, the initial set $E(0) \subset \mathbb {T}_R^3$ and let $E_1^{(h)}$ be a minimizer of (6.1) with $E(0) =E$. We construct the discrete-in-time evolution $(E_k^{(h)})_{k\in \mathbb {N}}$ as before by induction such that, assuming that $E_k^{(h)}$ is defined, we set $E_{k+1}^{(h)}$ to be a minimizer of (6.1) with $E= E_k^{(h)}$ and denote the associated potential for short by $U_{k+1}^{(h)}$, which is the solution of

6.5 $$\begin{aligned} -\Delta U_{k+1}^{(h)} = \frac{1}{h} \left( \chi _{E_{k+1}^{(h)}} - \chi _{E_{k}^{(h)}}\right) \end{aligned}$$

with zero average. The Euler-Lagrange equation now reads as

6.6 $$\begin{aligned} U_{k+1}^{(h)} = -\textrm{H}_{E_{k+1}^{(h)}} + \lambda _{k+1}^{(h)} \qquad \text {on }\, \partial E_{k+1}^{(h)}. \end{aligned}$$

Using the minimality of $E_{k+1}^{(h)}$ and comparing its energy against the previous set $E_k^{(h)}$, we obtain

6.7 $$\begin{aligned} P_{\mathbb {T}_R^3}(E_{k+1}^{(h)}) +\frac{h}{2} \mathfrak {D}(E_{k+1}^{(h)}, E_{k}^{(h)}) \le P_{\mathbb {T}_R^3}(E_{k}^{(h)}) , \end{aligned}$$

where $\mathfrak {D}(E_{k+1}^{(h)}, E_{k}^{(h)})$ is defined in (6.3).

As before we define the approximative flat flow $\{E^{(h)}(t)\}_{t \ge 0}$ by setting that

$$ E^{(h)}(t) = E_k^{(h)} \quad \text {and} \quad U^{(h)}(t) = U_k^{(h)} \quad \qquad \text {for }\, t \in [kh, (k+1) h) $$

and we call a *flat flow solution* of (1.8) any cluster point $\{E(t)\}_{t \ge 0}$ of $\{E^{(h)}(t)\}_{t \ge 0}$ as $h\rightarrow 0$, i.e.,

$$ E^{(h_n)}(t) \rightarrow E(t) \quad \text { in }L^1\text { for almost every }\, t >0 \, \text { and for some } \, h_n\rightarrow 0. $$

Arguing exactly as in [57, Proposition 3.1] we may conclude that there exists a flat flow solution of (1.8) starting from *E*(0) such that $P_{\mathbb {T}_R^3}(E(t)) \le P_{\mathbb {T}_R^3}(E(0))$, $|E(t)| = |E(0)|$ for every $t \ge 0$ and $\{E(t)\}_{t\ge 0}$ satisfies the equation (1.8) in a weak sense. Moreover the estimate (6.7) implies the dissipation inequality, which states that for all $T_2>T_1$ it holds that

6.8 $$\begin{aligned} \frac{1}{2}\int _{T_1+h}^{T_2+h} \Vert D\, U^{(h)}(t)\Vert _{L^2(\mathbb {T}_R^3)}^2 \, dt \le P_{\mathbb {T}_R^3}(E_{T_1}^{(h)}) - P_{\mathbb {T}_R^3}(E_{T_2}^{(h)}). \end{aligned}$$

Also the limit $U^{(h)}(t) \rightarrow U(t)$, up to a sub-sequence, exists for almost every *t*.

We state the following continuity result for the flat flow. The proof can be traced from [40], but we sketch it for the reader’s convenience.

### Lemma 6.1

Let $\{E^{(h)}(t)\}_{t \ge 0}$ be an approximative flat flow solution of (1.8) starting from a set of finite perimeter $E(0) \subset \mathbb {T}_R^3$ with volume $|E(0)| = v$ and perimeter $P_{ \mathbb {T}_R^3}(E(0))\le M$. Then for all $t>s\ge 2h$ with $h \le t-s\le 1$ it holds that

$$ |E^{(h)}(t) \Delta E^{(h)}(s)| \le C (t-s)^{\frac{1}{4}}, $$

where the constant depends on *v* and *M*.

### Proof

Let us fix *s*, *t* as in the assumptions and let $U^{(h)}(s)$ and $U^{(h)}(t)$ be the potentials associated with the sets $E^{(h)}(s)$ and $E^{(h)}(t)$. We use (6.4) and (6.8) for the approximative flat flow to deduce that

$$ \begin{aligned} \Vert \chi _{E^{(h)}(t)} -\chi _{E^{(h)}(s)}\Vert _{H^{-1}(\mathbb {T}_R^3)}&\le \int _{s-h}^{t+h} \frac{1}{h}\Vert \chi _{E^{(h)}(\tau +h)} - \chi _{E^{(h)}(\tau )}\Vert _{H^{-1}(\mathbb {T}_R^3)}\, d \tau \\&\le 2 \left( \int _{s-h}^{t+h}\frac{1}{h^2}\Vert \chi _{E^{(h)}(\tau +h)} -\chi _{ E^{(h)}(\tau )}\Vert _{H^{-1}(\mathbb {T}_R^3)}^2 \, d \tau \right) ^{\frac{1}{2}} \, \sqrt{t-s}\\&\le 2\left( \int _{h}^\infty \Vert D \, U^{(h)}(t)\Vert _{L^2(\mathbb {T}_R^3)}^2 \, d \tau \right) ^{\frac{1}{2}} \, \sqrt{t-s} \\&\le 2\sqrt{2 P_{\mathbb {T}_R^3}(E(0))} \sqrt{t-s}. \end{aligned} $$

By [40, Lemma 3.1], the following interpolation inequality holds for $\varphi \in BV(\mathbb {T}_R^3)$ and all $\rho \in (0,\rho _0)$:

6.9 $$\begin{aligned} \Vert \varphi \Vert _{L^1(\mathbb {T}_R^3)} \le \rho (\Vert D \varphi \Vert _{L^1(\mathbb {T}_R^3)} +c) + \frac{C}{\rho }\Vert \varphi \Vert _{H^{-1}(\mathbb {T}_R^3)}. \end{aligned}$$

We use (6.9) with $\varphi = \chi _{E^{(h)}(t) } - \chi _{E^{(h)}(s) }$ and have, by the above and by $P_{\mathbb {T}_R^3}(E^{(h)}({\bar{t}})) \le P_{\mathbb {T}_R^3}(E(0))$ for all ${\bar{t}} >0$, that

$$ \begin{aligned} |E^{(h)}(t) \Delta E^{(h)}(s)|&\le \rho ( P_{\mathbb {T}_R^3}(E(0)) + c) + \frac{C}{\rho } \Vert \chi _{E^{(h)}(t)} - \chi _{E^{(h)}(s)}\Vert _{H^{-1}(\mathbb {T}_R^3)}\\&\le \rho ( P_{\mathbb {T}_R^3}(E(0)) + c) + \frac{C}{\rho } \sqrt{2 P_{\mathbb {T}_R^3}(E(0))} \sqrt{t-s}. \end{aligned} $$

The claim follows by choosing $\rho = \rho _0(t-s)^{\frac{1}{4}}$. $\square$

To proceed, we need the following density estimate which is similar to [24, Proposition 4.1] and [60, Lemma 2.1]:

### Lemma 6.2

Let $E\subset \mathbb {T}_R^3$, with $R\ge 1$, be a set of class $C^3$, with $|E| = v$ and $P_{\mathbb {T}_R^3}(E) \le M$, and let $u_E \in C^1(\mathbb {T}_R^3)$ be a function with zero average such that $\Vert D u_E\Vert _{L^2(\mathbb {T}_R^3)} \le M$ and

6.10 $$\begin{aligned} \textrm{H}_E = -u_E +\lambda \quad \text {on } \, \partial E \quad \text { for some } \lambda \in \mathbb {R}. \end{aligned}$$

Then, for all $\rho \in (0,1),$ it holds that

6.11 $$\begin{aligned} \frac{1}{C} \le \frac{{\mathcal {H}}^2(\partial E \cap B_\rho (x))}{\rho ^2} \le C, \end{aligned}$$

where the constant $C>1$ depends only on *v* and *M*.

### Proof

We note that by (6.10) for every $X \in C^1(\mathbb {T}_R^3; \mathbb {R}^3)$ it holds that

6.12 $$\begin{aligned} \int _{\partial E} \textrm{div}^T X \, d {\mathcal {H}}^2 = \int _{E} \textrm{div}\big ((-u_E + \lambda )X\big ) \, dx. \end{aligned}$$

Therefore the statement follows from [60, Lemma 2.1] once we bound the Lagrange multiplier $\lambda \in \mathbb {R}$. The challenge is that we need to bound $\lambda$ such that the bound is independent of *R*.

To this aim we obtain by [25, Proposition 2.3], or [48, Lemma 2.1], that there is a radius $r \in (0,1)$ depending on *v*, *M*, and points $x_1, x_2 \in \mathbb {T}_R^3$ such that $|x_1 -x_2| > 4r$ and

6.13 $$\begin{aligned} |E\cap B_r(x_1)| \ge \frac{1}{2} |B_r| \qquad \text {and} \qquad |E\cap B_r(x_2)| \le \delta |B_r|, \end{aligned}$$

where $\delta >0$ is a small number which choice will be clear later. Let $\eta _\varepsilon$ be the standard convolution kernel and choose $f_1 = \chi _{B_{3r/2}(x_1)} * \eta _\varepsilon$ and $f_2 = 27 \chi _{B_{\frac{r}{2}}(x_2)} * \eta _\varepsilon$ for $\varepsilon = \frac{r}{4}$, and let $\zeta \in C^\infty (\mathbb {T}_R^3)$ be the solution of

6.14 $$\begin{aligned} \Delta \zeta = f_1 - f_2 \end{aligned}$$

in $\mathbb {T}_R^3$ with zero average.

Let us show that

6.15 $$\begin{aligned} \Vert D \zeta \Vert _{L^2(\mathbb {T}_R^3)} \le C \end{aligned}$$

for a constant *C*, which is independent of *R*. To this end, we multiply (6.14) by $\zeta$, integrate by parts, and recall the homogeneous Poincaré inequality in the three-dimension

$$ \Vert u\Vert _{L^6(\mathbb {T}_R^3)} \le C \Vert D u\Vert _{L^2(\mathbb {T}_R^3)}, $$

which holds for all *u* with zero average for a uniform constant *C*, and deduce that

$$ \begin{aligned} \Vert D \zeta \Vert _{L^2(\mathbb {T}_R^3)}^2&= - \int _{\mathbb {T}_R^3} \Delta \zeta \, \zeta \, dx = -\int _{\mathbb {T}_R^3} (f_1 - f_2) \, \zeta \, dx\\&\le \Vert f_1-f_2\Vert _{L^{\frac{6}{5}}(\mathbb {T}_R^3)} \Vert \zeta \Vert _{L^{6}(\mathbb {T}_R^3)} \le C \Vert D \zeta \Vert _{L^2(\mathbb {T}_R^3)}, \end{aligned} $$

where the constant depends on *v* and *M*. Hence we have (6.15).

Let us fix $x \in \mathbb {T}_R^3$. By standard estimates for the Poisson equation it holds, for $\alpha \in (0,1)$, that

$$ \Vert D^2 \zeta \Vert _{C^{\alpha }(B_1(x))} \le C( \Vert D \zeta \Vert _{L^2(B_2(x))} + \Vert f_1 - f_2\Vert _{C^{\alpha }(B_2(x))}), $$

where the constant depends only on $\alpha$. Since *x* is arbitrary, the bound (6.15) implies

6.16 $$\begin{aligned} \Vert D^2 \zeta \Vert _{L^\infty (\mathbb {T}_R^3)} \le C. \end{aligned}$$

We proceed by multiplying the equation (6.10) by $D \zeta \cdot \nu _E$, where $\nu _E$ is the outer unit normal of *E*, use the divergence theorem and have

$$\begin{aligned} \big |\int _{\partial E} (-u_E + \lambda )(D \zeta \cdot \nu _E) \, d {\mathcal {H}}^{2} \big |&=\big | \int _{\partial E} \textrm{H}_E (D\zeta \cdot \nu _E) \, d {\mathcal {H}}^{2} \big |=\big | \int _{\partial E} \text {div}^T D \zeta \, d {\mathcal {H}}^{2}\big | \\&\le C \Vert D^2 \zeta \Vert _{L^\infty } P_{\mathbb {T}_R^3}(E) . \end{aligned}$$

On the other hand, it holds that

$$ |\lambda | \big |\int _{\partial E} (D \zeta \cdot \nu _E) \, d {\mathcal {H}}^{2}\big | = |\lambda | \big |\int _{E} \Delta \zeta \, dx\big | = |\lambda | \big |\int _{E} f_1 - f_2\, dx\big |. $$

It follows from the choice of the points $x_1$ and $x_2$ in (6.13) that $\int _{E} f_1 - f_2\, dx \ge c_0>0$, for $c_0$ depending on *v* and *M*, when $\delta$ is small enough. Therefore, we need yet to show that

6.17 $$\begin{aligned} \big | \int _{\partial E} u_E(D \zeta \cdot \nu _E) \, d {\mathcal {H}}^{2}\big | \le C \end{aligned}$$

in order to have the uniform bound $|\lambda | \le C$.

We use the divergence theorem (for *E* and $\mathbb {T}_R^3 \setminus E$ ) and have

$$ \big | \int _{\partial E} u_E(D \zeta \cdot \nu _E) \, d {\mathcal {H}}^{2}\big | \le \frac{1}{2} \int _{\mathbb {T}_R^3} |u_E| |\Delta \zeta | + |D u_E| |D \zeta | \, dx. $$

By the assumption $\Vert D u_E\Vert _{L^2(\mathbb {T}_R^3)} \le M$ and by (6.15) we have that

$$ \int _{\mathbb {T}_R^3} |D u_E| |D \zeta | \, dx \le \Vert D u_E\Vert _{L^2(\mathbb {T}_R^3)} \Vert D \zeta \Vert _{L^2(\mathbb {T}_R^3)} \le C. $$

On the other hand, by the equation (6.14) and by the homogeneous Poincaré inequality it holds that

$$ \int _{\mathbb {T}_R^3} |u_E| |\Delta \zeta | \, dx \le \Vert u_E\Vert _{L^6(\mathbb {T}_R^3)} \Vert f_1 -f_2\Vert _{L^{\frac{6}{5}}(\mathbb {T}_R^3)} \le C \Vert D u_E\Vert _{L^2(\mathbb {T}_R^3)} \le C. $$

Hence we have (6.17), and the claim follows. $\square$

Since we have the density estimates we may use the trace theorem due to Meyers-Ziemer [44]. Indeed it follows from [44, Theorem 4.7] that if *E* is as in the statement of Lemma 6.2, and hence satisfies (6.11), then for every $\varphi \in C^1(\mathbb {T}_R^3)$ it holds that

6.18 $$\begin{aligned} \big | \int _{\partial E} \varphi \, d {\mathcal {H}}^2 \big | \le C' \Vert \varphi \Vert _{W^{1,1}(\mathbb {T}_R^3)}\,, \end{aligned}$$

with $C'$ depending on *M* and *R*. The following proposition then follows from Theorem 3.1 and Lemma 5.1:

### Proposition 6.3

Let $E \subset \mathbb {T}_R^3$ be $C^3$-regular set such that $|E|=|B_1|$, $P_{\mathbb {T}_R^3}(E) \le M$ and let $u_E \in C^1(\mathbb {T}_R^3)$ be a function with zero average which satisfies

$$ \textrm{H}_E = -u_E +\lambda \quad \text {on } \, \partial E \quad \text { for some } \lambda \in \mathbb {R}. $$

Then there exist $q \in (0,1)$ and $R_0= R_0(M)\ge 1$ such that when $R \ge R_0$, there are disjoint balls $B_{r}(x_1), \dots , B_{r}(x_N)$ with $r = N^{-\frac{1}{3}}$ such that for $F = \bigcup _{i=1}^N B_r(x_i)$ it holds that

$$ |E \Delta F| + |P(E) - 4 \pi N^{\frac{1}{3}}| \le C \Vert D u_E\Vert _{L^2( \mathbb {T}_R^3)}^q $$

where $C>1$ depends on *M* and *R*.

If in addition the balls $B_{r}(x_1), \dots , B_{r}(x_N)$ are quantitatively disjoint in the sense that $|x_i - x_j|\ge 2r +\delta _1$ with $i \ne j$ for some $\delta _1 >0$, then there is $\varepsilon = \varepsilon (\delta _1, M)>0$ such that if $|E \Delta F|\le \varepsilon$, where $F = \bigcup _{i=1}^N B_r(x_i)$, then the estimate improves as

$$ P(E) - 4 \pi N^{\frac{1}{3}} \le C \Vert D u_E\Vert _{L^2( \mathbb {T}_R^3)}^2, $$

for a constant *C* that depends on *M*, *R* and $\delta _1>0$.

### Proof

In order to prove the first inequality we may assume that $\Vert D u_E\Vert _{L^2( \mathbb {T}_R^3)} \le \varepsilon _1$, where $\varepsilon _1>0$ is a small number which choice will be clear later, since otherwise the claim is trivially true when we choose $F =B_1$ and the constant *C* large enough. Let us begin by proving that every component of the boundary $\partial E$ are quantitatively bounded. To be more precise if $\Sigma '$ is a component of $\partial E$ then we claim that it holds that

6.19 $$\begin{aligned} \text {diam}(\Sigma ') \le C \end{aligned}$$

for a constant that is independent of *R*. Indeed, this follows from the density estimate (6.11) by the following well-known argument. We can choose points $x_1, \dots , x_{k_0} \in \Sigma '$ such that $\frac{1}{2}\le |x_{i} - x_j|$ for $i \ne j$ and $k_0 \ge \text {diam}(\Sigma ')$. Then, by the perimeter bound and (6.11), it holds that

$$ M \ge {\mathcal {H}}^{2}(\Sigma ') \ge \sum _{i=1}^{k_0} {\mathcal {H}}^{2}(\Sigma ' \cap B_{\frac{1}{4}}(x_i)) \ge \frac{k_0}{16C} \ge \frac{\text {diam}(\Sigma ')}{16C} $$

and (6.19) follows. Thanks to the diameter bound (6.19) we may use the results from the previous sections when we choose $R_0$ large enough, i.e., $R_0 \ge 2C \ge 2 \text {diam}(\Sigma ')$.

We use (6.18) for $\varphi = u_E^2$ and obtain

$$ \Vert u_E\Vert _{L^2(\partial E)}^2 \le \Vert u_E^2 \Vert _{W^{1,1}(\mathbb {T}_R^3)} \le C' \Vert D u_E\Vert _{L^2(\mathbb {T}_R^3)}^2, $$

where the last inequality follows from Poincaré inequality. The assumption $\textrm{H}_E = -u_E +\lambda$ on $\partial E$ yields

6.20 $$\begin{aligned} \Vert \textrm{H}_E - \bar{\mathrm {{H}}}_E\Vert _{L^2(\partial E)}^2 \le \Vert \textrm{H}_E - \lambda \Vert _{L^2(\partial E)}^2 = \Vert u_E\Vert _{L^2(\partial E)}^2 \le C' \Vert D u_E\Vert _{L^2(\mathbb {T}_R^3)}^2. \end{aligned}$$

Since $\Vert D u_E\Vert _{L^2(\mathbb {T}_R^3)} \le \varepsilon _1$, then it holds $\Vert \textrm{H}_E - \bar{\mathrm {{H}}}_E\Vert _{L^2(\partial E)} \le \tau$, where $\tau >0$ is from Theorem 3.1, when $\varepsilon _1$ is small enough. Then Theorem 3.1 with (3.1) implies that

6.21 $$\begin{aligned} \sup _{x \in E \Delta F} |d_F| + |P_{\mathbb {T}_R^3}(E) - 4 \pi N^{\frac{1}{3}}| \le C \Vert \textrm{H}_E - \bar{\mathrm {{H}}}_E\Vert _{L^2(\partial E)}^q \le C' \Vert D u_E\Vert _{L^2(\mathbb {T}_R^3)}^q , \end{aligned}$$

where *F* is as in the statement. Hence we have the first claim.

In order to prove the second claim we again notice that we may assume that $\Vert D u_E\Vert _{L^2( \mathbb {T}_R^3)} \le \varepsilon _1$. The claim then follows from (6.19), (6.20) and from Proposition 5.1. $\square$

We are now ready the prove Theorem 1.3. Since the argument for the first statement is similar to [25, Theorem 1.1] (see also [17]), while the second statement follows from a similar argument as to that in the proof of Theorem 1.2, so we only give the outline of the proof.

### Proof of Theorem 1.3

Let $\{E^{(h_n)}(t)\}_{t\ge 0}$ be an approximative flat flow converging to $\{E(t)\}_{t\ge 0}$. As we discussed above, we may assume that $|E(0)| = |B_1|$.

Let us consider the first claim. We begin by using the dissipation inequality (6.8) for $T_l = l^2- h$, where $l = 1,2, \dots$ and obtain by the mean value theorem a sequence of times $T_l^h \in [l^2,(l+1)^2]$ such that

$$ \Vert D\, U^{(h)}(T_l^h)\Vert _{L^2(\mathbb {T}_R^3)}^2 \le \frac{1}{l}\int _{l^2}^{(l+1)^2} \Vert D\, U^{(h)}(t)\Vert _{L^2(\mathbb {T}_R^3)}^2 \, dt \le \frac{P_{\mathbb {T}_R^3}(E_0)}{l}. $$

Denote the associated sets by $E_l^h = E^{(h)}(T_l^h)$. Then by Proposition 6.3 we find an index $l_0$, which is independent of *h*, such that for every $l \ge l_0$ there is $N_l^h \in \mathbb {N}$ such that for the union of disjoint balls with radius $r_{l,h}= (N_l^h)^{-\frac{1}{3}}$, $F_l^h = \bigcup _{i=1}^{N_l^h} B_{r_{l,h}}(x_i^l)$, it holds that

6.22 $$\begin{aligned} |E_l^h \Delta F_l^h| + |P(E_l^h) - 4 \pi (N_l^h)^{\frac{1}{3}}| \le C\, l^{-\frac{q}{2}}. \end{aligned}$$

Since the perimeter is decreasing $$P(E^{(h)}(T_{l+1}^h)) \le P(E^{(h)}(T_{l}^h)) $$, also the number of the balls $$N_l^h $$ is decreasing for $$l\ge l_0$$. Therefore, by passing to the converging sub-sequence $$(h_n)$$, we obtain that the limit sequence $$\{N_l\}_{l \ge l_0}$$, where $$N_l^{h_n} \rightarrow N_l$$, is also monotone and thus it has a limit $$N = \lim _{l \rightarrow \infty } N_l$$ and there is $$l_1 \ge l_0$$ such that $$N_l = N$$ for all $$l \ge l_1$$. But since $$N_l^{h_n} \rightarrow N_l$$ and $$N_l^{h_n}$$ are also monotone, we deduce that for every $$L > l_1$$ it holds that

6.23 $$\begin{aligned} N_l^{h_n} = N \qquad \text {for all }\, l_1 \le l \le L \end{aligned}$$

when $$h_n $$ is small, depending on *L*.

We obtain from (6.22) and (6.23) that

$$ |P(E_l^{h_n}) - 4 \pi N^{\frac{1}{3}}| \le C\, l^{-\frac{q}{2}} \qquad \text {for all }\, l_1 \le l \le L $$

when $h_n$ is small. But again since $t \mapsto P(E^{(h_n)}(t))$ is monotone, we deduce that for every $\varepsilon >0$ there is $T_\varepsilon >1$, independent of $h_n$, such that, for all $t \in (T_\varepsilon -1, T)$ where $T > 2T_\varepsilon$ is arbitrary, it holds that

6.24 $$\begin{aligned} |P(E^{(h_n)}(t)) - 4 \pi N^{\frac{1}{3}}| \le \varepsilon , \end{aligned}$$

when $h_n\le h_0(\varepsilon ,T)$. Using this together with the dissipation inequality (6.8) we deduce that

$$ \frac{1}{2}\int _{T_\varepsilon }^{T} \Vert D \, U^{(h_n)}(t)\Vert _{L^2(\mathbb {T}_R^3)}^2 \, dt \le 2 \varepsilon . $$

Let us denote $I_\varepsilon ^{h_n} = \{ t \in (T_\varepsilon , T): \Vert D\, U^{(h_n)}(t)\Vert _{L^2(\mathbb {T}_R^3)}^2 \ge \sqrt{\varepsilon }\}$. Then by the above it holds that $|I_\varepsilon ^{h_n}| \le 4\sqrt{\varepsilon }$. Moreover by Proposition 6.3 we obtain that for all $t \in (T_\varepsilon , T) \setminus I_\varepsilon ^{h_n}$ there is a union of *N*-many disjoint balls with radius $r = N^{-\frac{1}{3}}$, $F^{(h_n)}(t) = \bigcup _{i=1}^{N} B_{r}(x_i^{h_n}(t))$ such that

$$ |E^{(h_n)}(t) \Delta F^{(h_n)}(t) | \le C \varepsilon ^{\frac{q}{4}}. $$

We use the Hölder continuity $|E^{(h_n)}(t) \Delta E^{(h_n)}(s)| \le C |t-s|^{\frac{1}{4}}$, for $s+h \le t \le 1$ from Lemma 6.1 and the fact that the above holds for all $t \in (T_\varepsilon , T) \setminus I_\varepsilon ^{h_n}$ with $|I_\varepsilon ^{h_n}| \le 4\sqrt{\varepsilon }$ to deduce that for all $t \in (T_\varepsilon ,T)$ it holds that

6.25 $$\begin{aligned} |E^{(h_n)}(t) \Delta F^{(h_n)}(t) | \le C \varepsilon ^{\frac{q}{8}}, \end{aligned}$$

where $F^{(h_n)}(t) = \bigcup _{i=1}^{N} B_{r}(x_i^{h_n}(t))$ for $r = N^{-\frac{1}{3}}$. The convergence $|E(t) \Delta F(t) |\rightarrow 0$ as $t \rightarrow \infty$ follows by passing (6.25) to the limit $h_n \rightarrow 0$ and recalling that *T* is arbitrary.

We proceed to prove the second claim. We assume still that $\{E^{h_n}(t)\}_{t\ge 0}$ is an approximate flat flow converging to $\{E(t)\}_{t\ge 0}$ and let *N*, $r>0$, and $x_i(t)$, $i=1, \dots , N$, be as in the statement. As in the proof of Theorem 1.2, we set that

$$ f_n(t) = P(E^{(h_n)}(t)), $$

and observe that the $f_n$’s are bounded monotone non-increasing functions. Therefore the functions $f_n$’s converge pointwise to some non-increasing function $f_\infty :[0,+\infty )\rightarrow \mathbb {R}$. It follows from (6.24) that $\lim _{t \rightarrow \infty } f_\infty (t) = 4 \pi N^{\frac{1}{3}}$. Again we distinguish two cases.

**Case 1.** Assume first $f_\infty (t)> 4\pi N^{\frac{1}{3}}$ for all $t\in [0, +\infty )$.

We recall that it follows from (6.25) that for any $\varepsilon >0$ there is $T_\varepsilon >1$ such that for all $t \in (T_\varepsilon , T)$, it holds that

$$ |E^{(h_n)}(t) \Delta F^{(h_n)}(t) | \le C \varepsilon ^{\frac{q}{8}}, $$

when $h_n$ is small. Here $F^{(h_n)}(t) = \bigcup _{i=1}^{N} B_{ r}(x_i^{h_n}(t))$ for $r = N^{-\frac{1}{3}}$ and $|x_i^{h_n}(t) - x_j^{h_n}(t)|> 2 r$. We use that fact that $|E^{(h_n)}(\cdot )\Delta E(\cdot )| \rightarrow 0$ uniformly in $[T_\varepsilon , T]$ to obtain

$$ |E(t) \Delta {\tilde{F}}(t) | \le C \varepsilon ^{\frac{q}{8}} \qquad \text {for all }\, t \in (T_\varepsilon , T), $$

where ${\tilde{F}}(t) = \bigcup _{i=1}^{ N} B_{ r}({\tilde{x}}_i(t))$. The assumption $|E(t) \Delta F(t) | \rightarrow 0$ as $t \rightarrow \infty$ for $F(t) = \bigcup _{i=1}^{ N} B_{r}(x_i(t))$ with $|x_i(t) -x_j(t)|\ge 2 r +\delta _1$ implies that, by possibly enlarging $T_{\varepsilon }$, we have

6.26 $$\begin{aligned} |E^{(h_n)}(t) \Delta F(t) | \le C \varepsilon ^{\frac{q}{8}} \qquad \text {for all }\, t\in (T_\varepsilon , T) \end{aligned}$$

when $h_n$ is small. We may thus use the second inequality in Proposition 6.3 to deduce

$$ P(E^{(h_n)}(t) ) - 4 \pi N^{\frac{1}{3}} \le C \Vert D\, U^{(h_n)}(t)\Vert _{L^2(\mathbb {T}_R^3)}^2 $$

for all $t \in (T_\varepsilon , T)$. We then use the assumption $f_\infty (t)> 4\pi N^{\frac{1}{3}}$ for all for all $t\in [0, +\infty )$ to deduce that it holds that

6.27 $$\begin{aligned} 0\le P(E^{(h_n)}(t) ) - 4 \pi N^{\frac{1}{3}} \le C \Vert D\, U^{(h_n)}(t)\Vert _{L^2(\mathbb {T}_R^3)}^2 \end{aligned}$$

for all $t \in (T_\varepsilon , T)$, when $h_n$ is small.

We proceed by choosing $k \ge \lfloor \frac{T_\varepsilon }{h_n}\rfloor +1$ and $k_T = \lfloor \frac{T}{h_n}\rfloor -1$. We use (6.7) iteratively and then (6.27) to obtain

$$ \begin{aligned} \frac{h_n}{2} \sum _{i = k}^{k_T-1} \mathfrak {D}(E_{i+1}^{(h_n)}, E_{i}^{(h_n)})&\le P_{\mathbb {T}_R^3}(E_{k}^{(h_n)}) -P_{\mathbb {T}_R^3}(E_{k_T}^{(h_n)}) \le P_{\mathbb {T}_R^3}(E_{k}^{(h_n)}) - 4 \pi N^{\frac{1}{3}} \\&\le C \Vert D\, U^{(h_n)}_{k}\Vert _{L^2(\mathbb {T}_R^3)}^2 = C \mathfrak {D}(E_{k}^{(h_n)}, E_{k-1}^{(h_n)}), \end{aligned} $$

where the equality follows from the definition (6.3) of the dissipation $\mathfrak {D}(\cdot , \cdot )$. We now argue precisely as in the proof of [24, Theorem 1.3] (which in turn is similar to the argument in the proof of Theorem 1.2) and deduce that for every $t \in (T_\varepsilon +h_n, T-h_n)$ it holds that

$$ h_n \sum _{i = \lfloor \frac{t}{h_n} \rfloor }^{\lfloor \frac{T}{h_n} \rfloor } \mathfrak {D}(E_{i+1}^{(h_n)}, E_{i}^{(h_n)}) \le C e^{- \frac{t}{C'}} $$

for a constants $C, C'$, depending on $M, \delta _1$ and *R*. Then, by (6.4) and by the above inequality we have that for $T_\varepsilon +h_n<s<t< s+1 < T-h_n$ it holds that

$$ \begin{aligned} \Vert \chi _{E^{(h_n)}(t)}&- \chi _{E^{(h_n)}(s)}\Vert _{H^{-1}(\mathbb {T}_R^3)} \le \sum _{i=\lfloor \frac{s}{h_n}\rfloor +1}^{\lfloor \frac{t}{h_n}\rfloor } \Vert \chi _{E^{(h_n)}_{i}} - \chi _{E^{(h_n)}_{i-1}}\Vert _{H^{-1}(\mathbb {T}_R^3)} \\&\le \frac{\sqrt{t-s}}{\sqrt{h_n}} \Big ( \sum _{i=\lfloor \frac{s}{h_n}\rfloor +1}^{\lfloor \frac{t}{h_n}\rfloor } \Vert \chi _{E^{(h_n)}_{i}} - \chi _{E^{(h_n)}_{i-1}}\Vert _{H^{-1}(\mathbb {T}_R^3)}^2\Big )^{\frac{1}{2}} \\&\le \frac{1}{\sqrt{h_n}} \Big ( \sum _{i=\lfloor \frac{s}{h_n}\rfloor +1}^{\lfloor \frac{t}{h_n}\rfloor } h_n^2 \, \mathfrak {D}(E^{(h_n)}_{i},E^{(h_n)}_{i-1})\Big )^{\frac{1}{2}}\\&\le C\, e^{-\frac{s}{2C'}}, \end{aligned} $$

when $h_n$ is small. Using (6.9) with $\varphi = \chi _{E^{(h_n)}(t)} - \chi _{E^{(h_n)}(s)}$ and $\rho = e^{-\frac{s}{4C'}}$, we obtain by the above that

$$ |E^{(h_n)}(t) \Delta E^{(h_n)}(s)| \le C\, e^{-\frac{s}{4C'}}, $$

by possible increasing *C*. Passing $h_n \rightarrow 0$ yields $|E(t) \Delta E(s)| \le C\, e^{-\frac{s}{4C_0}}$ for $T_\varepsilon< s<t< s+1<T$. Since *T* was arbitrary we deduce that *E*(*t*) converges exponentially fast to a set *F* which by (6.26) is of the form $F= \bigcup _{i=1}^{ N} B_{r}(x_i)$ with $|x_i -x_j| \ge 2 r +\delta _1$, i.e.,

$$ |E(t) \Delta F| \le C\, e^{-\frac{t}{4C'}}, $$

for *C*, which depends on *E*(0) and $\delta _1$. The convergence of the perimeters

$$ |P(E(t)) - 4 \pi N^{\frac{1}{3}} | \le C e^{-\frac{t}{C}} $$

follows from the same argument as in the proof of Theorem 1.2.

**Case 2.** There exists ${\bar{t}}>0$ such $f_\infty (t) = 4\pi N^{\frac{1}{3}}$ for all $t\in [{\bar{t}}, +\infty )$. In this case we argue exactly as in Theorem 1.2 to infer that $E(t) = F$ for every $t\ge {\bar{t}}$. $\square$

## Data Availability

No new data was created or analyzed in this study.
